# Lineage-specific co-evolution of the Egf receptor/ligand signaling system

**DOI:** 10.1186/1471-2148-10-27

**Published:** 2010-01-27

**Authors:** Juliette AGC Laisney, Ingo Braasch, Ronald B Walter, Svenja Meierjohann, Manfred Schartl

**Affiliations:** 1 Department of Physiological Chemistry I, Biocenter, University of Würzburg, Würzburg, Germany; 2 Department of Chemistry and Biochemistry, Texas State University, San Marcos, Texas, USA

## Abstract

**Background:**

The epidermal growth factor receptor (Egfr) with its numerous ligands has fundamental roles in development, cell differentiation and physiology. Dysfunction of the receptor-ligand system contributes to many human malignancies. Consistent with such various tasks, the Egfr gene family has expanded during vertebrate evolution as a consequence of several rounds of whole genome duplication. Of particular interest is the effect of the fish-specific whole genome duplication (FSGD) on the ligand-receptor system, as it has supplied this largest group of vertebrates with additional opportunities for sub- and/or neofunctionalization in this signaling system.

**Results:**

We identified the predicted components of the Egf receptor-ligand signaling system in teleost fishes (medaka, platyfish, stickleback, pufferfishes and zebrafish). We found two duplicated *egfr *genes, *egfra *and *egfrb*, in all available teleost genomes. Surprisingly only one copy for each of the seven Egfr ligands could be identified in most fishes, with zebrafish *hbegf *being the only exception. Special focus was put on medaka, for which we more closely investigated all Egf receptors and Egfr ligands. The different expression patterns of *egfra*, *egfrb *and their ligands in medaka tissues and embryo stages suggest differences in role and function. Preferential co-expression of different subsets of Egfr ligands corroborates the possible subfunctionalization and specialization of the two receptors in adult tissues. Bioinformatic analyses of the ligand-receptor interface between Egfr and its ligands show a very weak evolutionary conservation within this region. Using *in vitro *analyses of medaka Egfra, we could show that this receptor is only activated by medaka ligands, but not by human EGF. Altogether, our data suggest a lineage-specific Egfr/Egfr ligand co-evolution.

**Conclusions:**

Our data indicate that medaka Egfr signaling occurs via its two copies, Egfra and Egfrb, each of them being preferentially coexpressed with different subsets of Egfr ligands. This fish-specific occurrence of Egf receptor specialization offers unique opportunities to study the functions of different Egf receptor-ligand combinations and their biological outputs in vertebrates. Furthermore, our results strongly support the use of homologous ligands in future studies, as sufficient cross-specificity is very unlikely for this ligand/receptor system.

## Background

Signaling by the epidermal growth factor receptor (Egfr, also named ErbB1 or HER in humans) has fundamental roles in mammalian development, where it regulates diverse processes such as eyelid opening, tooth growth, wound healing, hair follicle and mammary gland development. On the cellular level, it controls key functions including cell division, differentiation, survival, motility and apoptosis [1]. Thus, it is not surprising that many tumors are associated with EGF receptor overexpression, mutations or autocrine production of growth factors [2,3].

The use of animal models such as *Drosophila melanogaster *and *Caenorhabditis elegans *has provided considerable advancement in understanding Egf receptor functions in development and physiology [4-6]. In vertebrate model organisms, numerous studies have been conducted in the mouse, but in the common fish models - the zebrafish (*Danio rerio*) and the Japanese medaka (*Oryzias latipes*) - only few publications address Egfr function. In zebrafish, Egf receptor signaling was shown to regulate cardiovascular processes during development [7]. Furthermore, it promotes oocyte maturation *in vitro *[8] and *in vivo *[9]. Egf receptor signaling appears to be self-regulated by several of its ligands in the zebrafish ovarian follicle [10], as deduced from the usage of recombinant human (rh) EGF, rh-betacellulin (BTC) and rh-heparin-binding EGF-like growth factor (HBEGF). For the medaka, Egfr detection with a human antibody directed against the tyrosine kinase domain showed temporarily and spatially different expression patterns during development [11].

It is generally accepted that in addition to the two whole genome duplication events that occurred early in the vertebrate lineage ("R1" and "R2 duplication"), a later third whole genome duplication event, the so-called FSGD (fish-specific genome duplication or R3) occurred within the actinopterygian (ray-finned) fish lineage approximately 320-350 million years ago [12-14]. Most of the duplicated genes have been lost secondarily (nonfunctionalization). In many instances, however, gene duplicates from this event have persisted within fish genomes, for example when one of the duplicates acquired a new function (neofunctionalization) and/or when the ancestral gene functions have been distributed among the two gene copies (subfunctionalization) [14-16].

In the present study, we carried out phylogenetic and synteny analyses for a detailed insight into the evolution of Egf receptors and their ligands in teleost fishes.

Importantly, all previous studies in fish (zebrafish, goldfish and trout) that included the application of Egf receptor ligands were performed using recombinant mammalian proteins [9,17,18]. This implies that mammalian Egfr and Egfr ligands share high structural similarity and functional homology with their fish counterparts and that mammalian ligands consequently have the ability to interact with the fish Egfr binding domain to trigger its dimerization and activation. However, it is unclear whether heterologous application of ligands truly mimics the physiological situation. Therefore, we examined Egf receptor domains conservation between tetrapods and teleosts and investigated the domains involved in ligand binding. We particularly focused on medaka, monitored expression pattern profiles for the two copies of the Egf receptor and the seven Egf receptor ligands in the embryo and adult fish. Finally, we developed an *in vitro *system to verify the functionality of medaka Egf receptor ligands in terms of receptor activation. To our knowledge, this is the first work encompassing a functional analysis of the fish Egf receptor-ligands system in this evolutionary context.

## Results and Discussion

### Phylogenetic and syntenic analyses of teleost Egfr ligands

In mammals, seven ligands bind and activate Egfr, namely epidermal growth factor (Egf), transforming growth factor alpha (Tgfa), amphiregulin (Areg), betacellulin (Btc), epiregulin (Ereg), heparin-binding EGF-like growth factor (Hbegf) and epigen (Epgn).

Using BLASTn and tBLASTn searches, we thoroughly surveyed the teleost genome assemblies from medaka (*Oryzias latipes*), stickleback (*Gasterosteus aculeatus*), green spotted pufferfish (*Tetraodon nigroviridis*), torafugu (*Takifugu rubripes*) and zebrafish (*Danio rerio*) http://www.ensembl.org/ - as well as a platyfish (*Xiphophorus maculatus*) EST sequence database (R. B. Walter et al., unpublished) for Egf receptors and Egf receptor ligands. While we could identify two gene copies for the Egf receptor in all teleost species investigated, surprisingly only one gene copy for each of the seven Egfr ligands was isolated except for zebrafish that possesses two gene copies of the *hbegf *gene. A recent study by Kassahn et al. in five teleost fishes [15] also provided evidence that duplicated Egfr but not Egfr ligands genes have been retained after FSGD. Amino acid sequences alignments of the identified tetrapod and teleost Egf receptors and of the mature Egfr ligands are shown in [Additional file 1: Supplemental figure S1B] and [Additional file 2].

**Figure 1:**
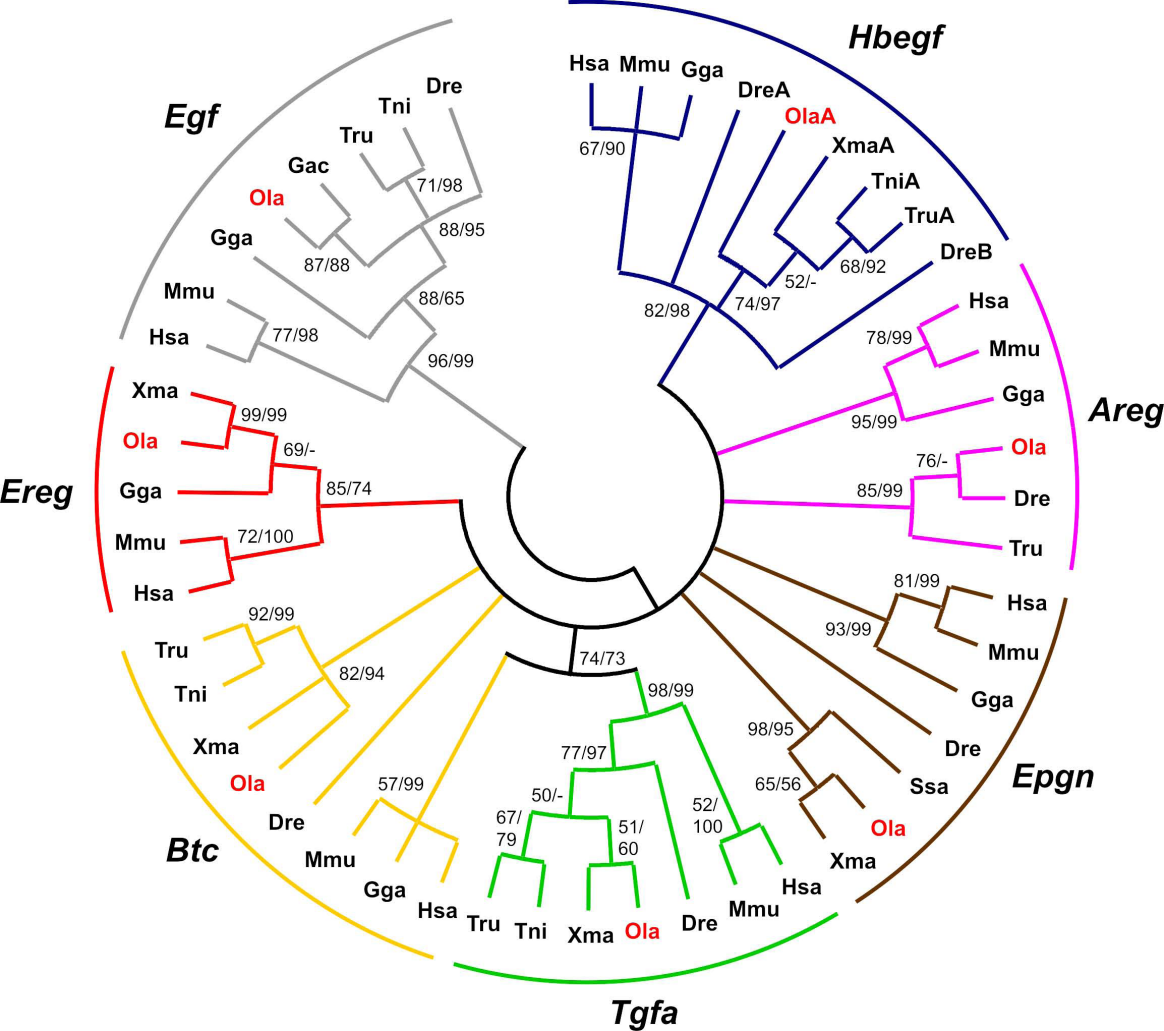
**Phylogeny of Egfr ligand genes**. Circle tree representation of a Maximum Likelihood consensus tree based on 138 bp of nucleotide sequences encoding the Egf domain (GTR+G+I substitution model). Bootstrap values for Maximum Likelihood and Neighbor Joining methods are shown. The tree was rooted on the branch leading to *Egf *sequences. Monophyly is supported for *Egf*, *Hbegf*, *Tgfa *and *Ereg *genes, but many other nodes remain unresolved [Additional file 10: Supplemental table S3]. Dre, *Danio rerio*; Gac, *Gasterosteus aculeatus*; Gga, *Gallus gallus*; Hsa, *Homo sapiens*; Mmu, *Mus musculus*; Ola (red), *Oryzias latipes*; Ssa, *Salmo salar*; Tni, *Tetraodon nigroviridis*; Tru, *Takifugu rubripes*; Xma, *Xiphophorus maculatus*.

**Figure 2:**
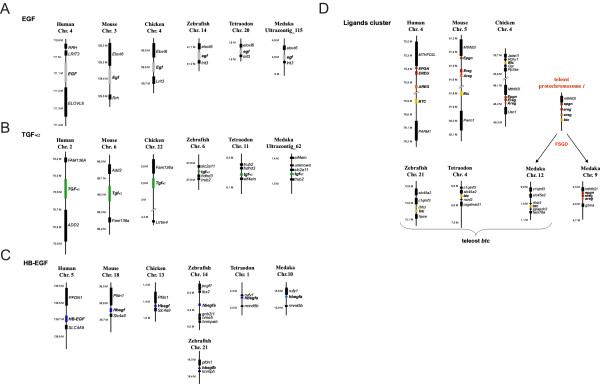
**Synteny analysis of vertebrate *Egf*, *Tgfa*and *Hbegf *and ligand cluster regions**. A) *Egf *region. B) *Tgfa*region. C) *Hbegf *region. D) *Epgn*, *Ereg*, *Areg *and *Btc*region.

For a more detailed insight into Egfr ligand evolution, a phylogenetic analysis based on tetrapod and teleost Egfr ligand genes was performed. The mature part of the ligands containing the Egfr-interacting motif was found to be the only part sufficiently conserved among all species to allow a phylogenetic tree reconstruction. A maximum likelihood (ML) bootstrap consensus tree of the 138 bp nucleotide sequences encoding the mature part of the ligands is shown in Figure 1. A ML tree based on the original data is shown in [Additional file 3]. Due to the short alignment used for its reconstruction, the trees exhibit many collapsed or poorly supported nodes, respectively. Based on these and other trees obtained with different methods (not shown), the orthology between teleost and tetrapod genes is well supported only for *Egf*, *Tgfa, Hbegf*, and *Ereg*. Therefore, we also undertook micro- and macro-synteny conservation analyses (Figure 2 and [Additional file 4]) in order to obtain further information about the relationship of the teleost ligands to their tetrapod counterparts.

### The Egf ligand gene

The chromosomal region surrounding the *egf *gene showed conserved synteny between and within tetrapods and teleosts (Figure 2A). Moreover, in *D. rerio *and *T. nigroviridis*, the *egf *gene is located on chromosomes Dre14 and Tni20, which have been shown to correspond to the *EGF *containing human chromosome Hsa4 [19]. A further large-scale synteny conservation analysis using the Synteny Database [20,21] revealed that the *egf*-containing regions in *D. rerio *[Additional file 4: Supplemental figure S4A] and *T. nigroviridis *[Additional file 4: Supplemental figure S4B] are derived from the teleost protochromosome *f *[19,22]. Medaka *egf *has not been mapped to any chromosome yet and is found on ultracontig 115. Still it was found in the vicinity of the same genes that surround *EGF *in the human genome (Figure 2A). In addition, the phylogenetic grouping with characterized tetrapod and zebrafish *egf *(Figure 1) and the gene's unique structure further validated it as true *EGF *ortholog. From the large-scale synteny analysis it can be inferred that medaka *egf *is most likely located on chromosomes Ola10 or Ola18 [Additional file 4: Supplemental figure S4C].

### The Tgfa ligand gene

*Tgfa *displayed no conserved synteny between tetrapods and teleosts (Figures 2B and [Additional file 4: Supplemental figure S5]). *D. rerio *and *T. nigroviridis tgfa *are located on chromosomes Dre6 and Tni11 respectively, which possess only few orthologous genes that are located on *TGFA *containing human chromosome Hsa2 [23,24] [Additional file 4: Supplemental figure S5A and S5B]. Medaka *tgfa *(ultracontig 62) has not been mapped to a particular chromosome. The large-scale analysis shows that the *TGFA *region on Hsa2 is syntenic to several chromosomes in the three teleost genomes but not to the *tgfa *containing ones [Additional file 4: Supplemental figure S5A to S5C]. This might be due to an interchromosomal translocation of the *tgfa *gene in the teleost ancestor. Nevertheless, the high bootstrap values of the teleost and tetrapod node (see Figure 1), support the orthology of vertebrate *Tgfa *genes.

### The Hbegf ligand gene

In the micro-synteny analysis, no conservation of synteny in *Hbegf *regions was found between tetrapods and teleosts. The gene order is only maintained within the group of tetrapods or teleosts independently (Figure 2C). However, teleost *hbegf *genes are located on Dre14, Dre21, Tni1 and Ola10, which were reported previously to be syntenic to Hsa5 where the *HBEGF *gene is found [19,23,24] [Additional file 4: Supplemental figure S6]. In addition, the phylogeny data also support the grouping of teleost *hbegf *with tetrapod *Hbegf *(Figure 1).

The zebrafish is the only investigated teleost species that has two paralogs for *hbegf*. One copy, called *hbegfa*, is located on chromosome Dre14 (orthologous to Ola10 *hbegf*); the other, called *hbegfb*, is found on Dre21 (corresponding to Ola14). The macro-synteny analysis revealed that the region containing *HBEGF *on Hsa5 is highly syntenic to Dre14 and Dre21, Tni1 and Tni7, as well as Ola10 and Ola14 [Additional file 4: Supplemental figure S6]. These chromosomes were previously shown to be derived from the teleost protochromosome *g *[19,22]. Thus, the most-likely scenario is that *hbegf *gene was initially duplicated in the teleost ancestor during the FSGD event. While *hbegfa *and *hbegfb *were kept in zebrafish, only the *hbegfa *copy was maintained in medaka and pufferfish, and the *hbegfb *copy was lost. So far, the two zebrafish *hbegf *paralogs are the only known teleost Egfr ligand genes that were maintained in two copies after the fish specific genome duplication.

### The Epgn-Ereg-Areg-Btc ligand gene cluster

An interesting feature of the Egfr ligands is the clustering of the four remaining ligand genes, namely *Epgn*, *Ereg*, *Areg*and *Btc *on human Hsa4 and mouse Mmu5 (Figure 2D). This cluster was also found on all well-annotated mammalian genomes. In chicken, only *Epgn*, *Ereg *and *Areg *genes are part of a cluster on Gga4. Two exons of chicken *Btc *could be found on another segment of Gga4 but they were not defined well enough to conclude the presence of a full functional *Btc *on chromosome 4 in this species. Still, it is likely that birds also possess a functional *Btc *gene, since it was possible to identify a well defined *Btc *gene on chromosome 4 of the zebrafinch (*Taeniopygia guttata*) (data not shown).

The orthology of these tetrapod genes with their teleost counterparts could not be resolved from the phylogenetic tree as most of the nodes were unresolved or poorly supported. The only exception is the relatively well supported monophyly of vertebrate *Ereg *genes (Figure 1). We note that a previous study by Stein and Staros [25] described similar problems with establishing the relationships of these genes among vertebrates using phylogenetic reconstructions only. Therefore, we relied here on conservation of synteny to clarify the orthology of the respective medaka genes.

In medaka three of the ligands are also present in a cluster on Ola9, namely *epgn*, *ereg *and *areg *(Figure 2D). The medaka *btc *gene is located on Ola12. In contrast to that, *epgn *was not found in pufferfishes and no *ereg *gene could be identified in zebrafish and pufferfish genomes. The zebrafish *areg *(Dre5) and *epgn *(Dre8) genes showed no conserved microsynteny with the medaka cluster (data not shown). However, there is conserved synteny between the teleost *btc *regions (Dre21, Tni4 and Ola12) (Figure 2D).

According to the macro-synteny analysis, Dre5, Dre8 and Dre21, Tni12 and Tni4, as well as Ola9 and Ola12 share numerous orthologous genes with the ligand cluster region on Hsa4 [Additional file 4: Supplemental figure S7]. These teleost chromosomes were previously reported to be derived from the teleost protochromosome *i *[19,22]. Thus, it appears that the ligand cluster located on protochromosome *i *was duplicated during the FSGD. After the FSGD, one of the cluster copies, which is now present on Ola9, has lost *btc*. In the zebrafish lineage, it broke further apart, separating *areg *and *ereg *genes. The other cluster then lost all genes but *btc *now found on Ola12, Tni4 and Dre21, respectively (Figure 2D). Thereby, the single teleost *epgn-ereg-areg *and *btc *genes are located on paralogous rather than orthologous chromosomal segments. Thus, the absence of the complete cluster in teleosts is not the result of translocation of *btc *to a new chromosome, but is due to differential gene loss from initially two clusters after the FSGD. A similar phenomenon has been observed for example for the Parahox cluster [26,27].

### Evolution of the vertebrate Egfr ligand gene family by whole genome and other types of duplications

**Figure 3:**
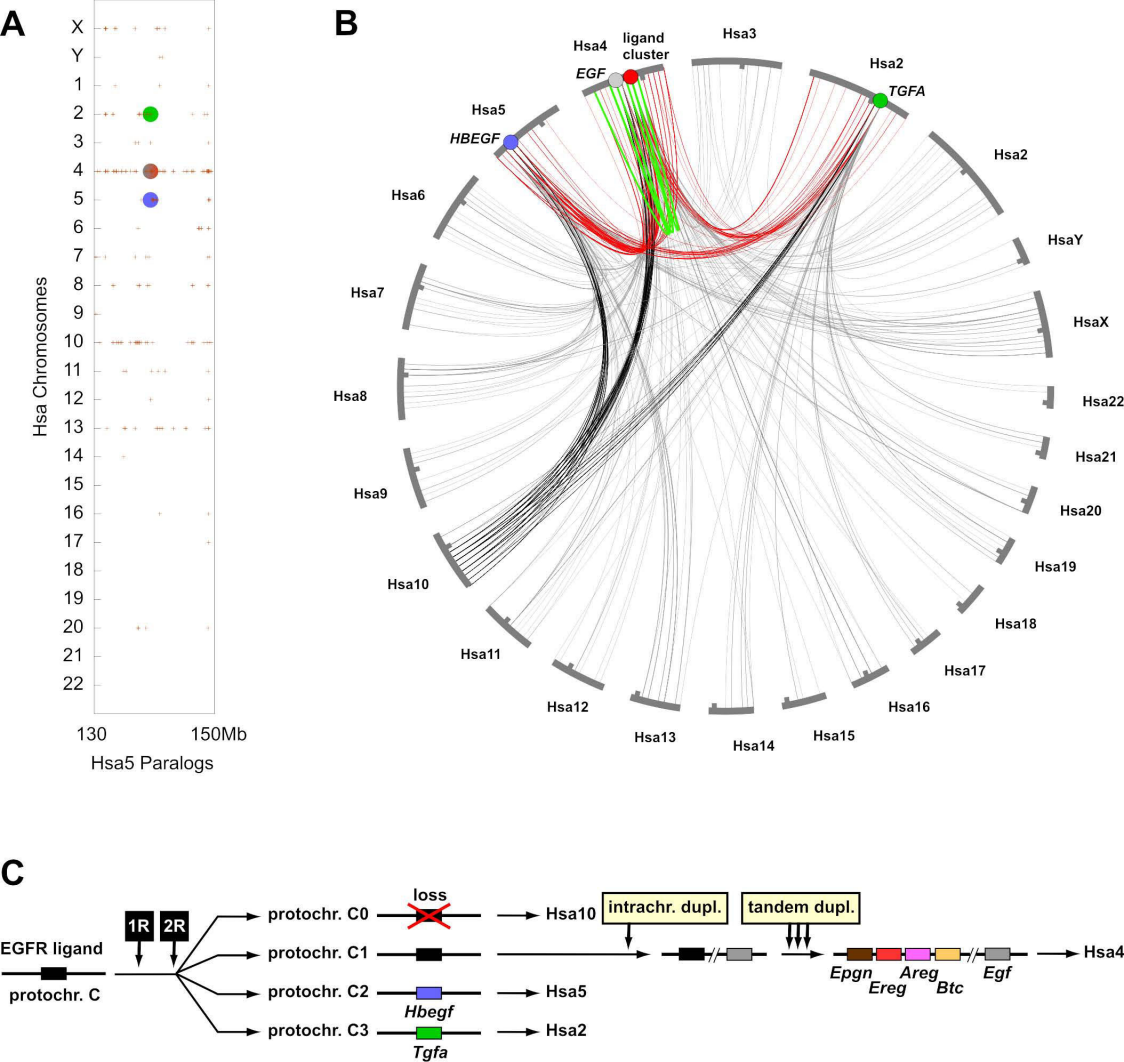
**Synteny and evolution of human EGFR ligand regions**. A) The surroundings of human EGFR ligand genes were analyzed with the Synteny Database [21]. Dot plots of paralogous genes in the human genome are displayed in red. The *HBEGF *gene region (blue circle) on Hsa5 was used as reference. Paralogs are indicated by red crosses and are found around the other EGFR ligand regions on Hsa2 (*TGFA*, green circle) and Hsa4 (*EGF *and ligand cluster, gray-red circle). Chromosomes Hsa10 and to a lesser degree also Hsa13 and Hsa8 share many paralogous connections with the *HBEGF *region. Similar pictures were obtained using other EGFR ligand regions as reference. B) Circle plot of paralogous genes from the human EGFR ligand regions as obtained from the Synteny Database[21]. Many paralogous connections are found between the EGFR ligand regions (red lines). All four regions also share many paralogous connections with Hsa10 (black lines). On Hsa4, several intrachromosomal paralogous connections (green lines) are found. C) Model of Egfr ligand gene family evolution in vertebrates. An ancestral gene on vertebrate protochromosome *C *was duplicated twice during the R1 and R2 whole genome duplications. The gene on gnathostome protochromosome *C0 *was lost, while the gene on protochromosome *C1 *was duplicated intrachromosomally. One of the two descendent genes became *Egf*, the other one was duplicated in tandem giving rise to the ligand cluster. The protochromosome nomenclature follows ref [22].

How did the Egfr ligand gene family in vertebrates evolve in the first place? To answer this question, the surroundings of the human EGFR ligand genes were analyzed with the Synteny Database [20,21]. Dot plots of paralogous genes within the human genome revealed that the human EGFR ligand regions on Hsa2 (*TGFA*), Hsa4 (*EGF *and ligand gene cluster) and Hsa5 (*HBEGF*) are highly syntenic to each other as well as with Hsa10, which does not contain an EGFR ligand gene (Figure 3A). This pattern also became visible using a circle plot representation of human EGFR ligand regions: all four EGFR ligand gene regions share many paralogous connections with each other (Figure 3B). Furthermore, all regions are connected to Hsa10, while no other human chromosomes shares comparable numbers of paralogous connections with all four EGFR ligand regions.

It has been previously hypothesized that parts of chromosomes Hsa2, Hsa4, Hsa5 and Hsa10 are derived from the vertebrate protochromosome *C*, which was then duplicated twice during the R1/R2 whole genome duplications giving rise to the gnathostome protochromosomes *C0*, *C1*, *C2 *and *C3*[22]. The chromosomal locations of Egfr ligand genes in the chicken and teleost genomes (Figure 2) are also compatible with being derived from vertebrate protochromosome *C *and its four gnathostome derivatives. Thus, we hypothesize that all Egfr ligand genes in the present vertebrate genomes can be traced back to a single pre-R1/R2 Egfr ligand gene. Importantly, this does not rule out the possibility that multiple Egfr ligand genes were present before the R1/R2 genome duplications. Our model rather suggests that only one gene gave rise to the diversity of Egfr ligand genes found in vertebrate genomes today. The situation in other chordates, which could help to resolve this issue, remains unclear as we did not find clear orthologs of the vertebrate Egfr ligand in the genomes of *Ciona *or amphioxus using BLAST and synteny analyses (data not shown).

According to our model (Figure 3C), the ancestral Egfr ligand gene was then duplicated twice during the R1/R2 genome duplications. The Egfr ligand gene located on gnathostome protochromosome *C0*, which later became part of Hsa10, was lost secondarily. Protochromosome *C1*gave rise to Hsa4 [22]. The Egfr ligand gene on this protochromosome was duplicated again onto the same chromosome. This might have been the duplication of a larger chromosomal block as several paralogous connections are found between the two EGFR ligand gene regions on Hsa4. One of the two genes then became *Egf*, while the other gene underwent several tandem duplications, thereby generating the EGFR ligand gene cluster. The fact that the *egf *and cluster genes are not found on the same chromosome in teleosts may be due to the fission of protochromosome *C1 *in fish [22].

Our model suggests that an original cluster consisting of *Epgn*, *Ereg*, *Areg*and *Btc *dates back at least to a common bony vertebrate ancestor of fish and tetrapods. Unfortunately, the genome assemblies of elephant shark (cartilaginous vertebrates) and lamprey (jawless vertebrates) are still too fragmentary to get more insights into the timing of the Egfr ligand cluster establishment in vertebrates (data not shown). Since its establishment, the cluster linkage has then been kept in the tetrapod lineage but is absent in the teleost lineage due to the differential loss of gene duplicates after the FSGD (see above).

Protochromosomes *C1 *and *C3*, finally, became parts of Hsa5 and Hsa2 containing *HBEGF *and *TGFA*, respectively.

We note that other ligand genes with Egf domain, i.e. tomoregulins, neuregulins and others, may be related to the canonical seven Egfr ligands analyzed here [25,28]. However, the tomoregulin genes do not show obvious synteny with the Egfr ligand gene regions. Their origin in relation to the canonical Egfr ligands will have to be further investigated. The neuregulin gene family members (*NRG1*, *NRG2*, *NRG3*), in contrast, are also located in protochromosome *C*-derived regions (Hsa8, Hsa5, Hsa13) and could have evolved along with the canonical Egfr ligands (data not shown).

### Evolution of tetrapod and teleost Egfr subdomains

To get a better insight into the evolutionary need of maintaining so many Egfr ligands, and, at least in fish, two copies of Egfr, we investigated the conservation of the functional domains of the receptor, including the receptor-ligand interface. It is generally assumed that functional and interaction sites *e.g*. ligand binding pockets, are usually under high selection pressure and therefore more conserved than other subdomains of the protein. We investigated here the variability of functional sites of tetrapod and teleost Egfr homologs, with a special focus on the receptor-ligand protein interface.

#### Similarity between tetrapod and teleost Egfr subdomains

The epidermal growth factor receptor is a transmembrane receptor tyrosine kinase [29]. It comprises an extracellular domain (ECD), a transmembrane domain (TM), an intracellular juxtamembrane domain (JM) and a tyrosine kinase domain (TK) [Additional file 1: Supplemental figure S1A].

For our evolutionary study we looked at the amino acid conservation for each domain of the EGF receptor between tetrapod and teleost species (Table 1). All teleost species we investigated possess two gene copies for the Egf receptor gene, *egfra *and *egfrb*, with *egfra *being more similar to tetrapod *Egfr*. For our amino acid analysis, we omitted the domain located carboxy-terminally of the kinase domain since its length is highly divergent between the investigated species. Nevertheless, the alignment between human EGFR and medaka Egfra and Egfrb displayed in [Additional file 5] shows that all major phosphorylation sites of the carboxy-terminal tail are conserved.

**Table 1:** Amino acid similarity between tetrapod and teleost EGF receptor subdomains

***Extra-cellular domain***									
	Hsa	Mmu	Gga	Ola	Xma	Dre	Tni	Tru	Gac
Hsa	100								
Mmu	88	100							
Gga	72	72	100						
Ola	57	56	57	100					
Xma	55	54	56	80	100				
Dre	58	56	58	66	63	100			
Tni	55	57	57	75	74	64	100		
Tru	55	55	57	79	76	66	86	100	
Gac	55	55	57	80	75	65	75	79	100
**Trans- and Juxta-membrane**									
	**Hsa**	**Mmu**	**Gga**	**Ola**	**Xma**	**Dre**	**Tni**	**Tru**	**Gac**
**Hsa**	**100**								
**Mmu**	91	**100**							
**Gga**	85	88	**100**						
**Ola**	74	72	77	**100**					
**Xma**	74	72	75	90	**100**				
**Dre**	80	81	82	81	82	**100**			
**Tni**	75	74	78	91	94	85	**100**		
**Tru**	75	74	78	90	94	87	97	**100**	
**Gac**	75	74	78	90	91	84	95	94	**100**

**Tyrosine kinase domain**									
	**Hsa**	**Mmu**	**Gga**	**Ola**	**Xma**	**Dre**	**Tni**	**Tru**	**Gac**
**Hsa**	**100**								
**Mmu**	98	**100**							
**Gga**	97	97	**100**						
**Ola**	90	90	91	**100**					
**Xma**	90	90	91	96	**100**				
**Dre**	89	89	91	95	94	**100**			
**Tni**	88	88	88	91	93	93	**100**		
**Tru**	91	91	92	96	97	95	95	**100**	
**Gac**	91	91	92	94	95	95	94	97	**100**

Similarity percentages between human (Hsa), mouse (Mmu), chicken (Gga), medaka (Ola), platyfish (Xma), zebrafish (Dre), green-spotted pufferfish (Tni), fugu (Tru) and three-spined stickleback (Gac) Egfr extracellular, trans- and juxta-membrane and intracellular tyrosine kinase domains.

Expectedly, the tyrosine-kinase domain displays the highest similarity between tetrapod Egfr and teleost Egfra (88 to 92%, Table 1). The intracellular juxtamembrane domain, involved in the allosteric control of ligand binding by stabilization of the tyrosine kinase domain dimer [30-32], is also well conserved. Conversely, the whole extracellular domain is the least conserved part of the receptor, even when compared among teleosts (55 to 58% similarity).

Three-dimensional structures of the extracellular domains (ECD) have been resolved for human EGFR, revealing four subdomains in the extracellular part of the receptor [33,34]. Subdomains I and III are involved in the formation of high-affinity ligand binding pocket, subdomains II and IV are forming an intramolecular autoinhibitory tether in the absence of ligand. On this level, subdomains I and III, implicated in ligand binding, exhibit only low identity between tetrapods and teleosts (47-61%) [Additional file 6: Supplemental table S1]. Subdomain II, involved in receptor dimerization, is the most conserved part of the extracellular domain with 62 to 67% identity. Presumably, this is at least partly due to the numerous conserved cysteine residues necessary for the proper secondary structure.

Altogether, this indicates that the Egfr extracellular part may have evolved differently compared to the rest of the protein between tetrapods and teleosts, and even among teleosts.

#### The Egfr/Egfr ligand interface

We were particularly interested in the evolutionary conservation of subdomains I and III, which compose the interface between receptor and ligand. To analyze this region in detail, we compared the human EGFR residues known to directly interact with EGF and TGFA [33,34] with their corresponding counterparts in medaka Egfra and Egfrb [Additional file 7]. We analyzed these receptor regions using the ConSurf tool http://consurf.tau.ac.il/[35]. This tool calculates evolutionary conservation scores between homologous proteins from different species and maps them on a given three-dimensional protein structure. Thus, important regions on the protein surface can be identified and visualized. The conservation score of a given amino acid residue is directly linked to its evolutionary rate. Some sites evolve slowly and are referred to as "conserved" while others evolve rapidly and are referred to as "variable".

As input, we supplied protein alignment and phylogenetic tree of human, mouse, rat and chicken Egfr and their medaka, platyfish, spotted green pufferfish, torafugu and stickleback Egfra and Egfrb co-orthologous proteins. The three-dimensional structure of the extracellular part of human EGFR in complex with TGFA [33] was chosen as template. The overall view [Additional file 7: Supplemental figure S9A] outlines the three main interaction sites between receptor and ligand. [Additional file 7: Supplemental figure S9B and S9C] display the interface between receptor and ligand in more detail. [Additional file 7: Supplemental figure S9D] recapitulates all amino acid substitutions between human and teleost species for Egfr residues that directly interact with Egfr ligands within subdomains I and III.

Amino acid substitution can either be classified as radical or conservative, whether it involves or not a major change in the amino acid physicochemical properties. Our analyses reveal that many amino acids located in the ligand-binding pocket are not conserved among vertebrates, and some even replaced with residues that induce a radical amino acid substitution. A similar picture emerged when comparing the Egfr ligands, namely tetrapod and teleost Egf and Tgfa [Additional file 2]. In both cases, amino acids that directly interact with Egfr [33,34] are not always conserved between species. Examples are the non-polar Met17 in mammalian Egf that is replaced by the small polar Asp in chicken and by the aromatic Phe in teleosts. Likewise, the aliphatic Ile19 in mammals is replaced by a smaller aliphatic Val residue in chicken and by the aromatic Phe or Tyr in teleosts. Another example is the positively charged Lys24 in human EGFR that is replaced by a small polar Ser in mouse, a small polar Asp in chicken, small non-polar Ala in medaka and small polar Ser in all other investigated teleosts and the mouse.

This lack of conservation suggests separate ways of receptor-ligand co-evolution in tetrapods and teleosts. It also implicates a possible subfunctionalization of the duplicated teleost receptors. To further investigate this hypothesis, we analyzed receptor and ligand expression exemplarily for teleosts in the medaka fish.

### Expression of medaka *egfrs *and Egfr ligands

#### Embryonic expression

Both *egfra *and *egfrb *transcripts are present from stage 8 (before mid-blastula transition) to the hatching stage, but with much lower levels of expression past the stage 16 (late gastrula stage) when compared to stage 8 [Additional file 8: Supplemental figure S10A]. This suggests that both receptor transcripts are maternally deposited in the embryo and low zygotic expression levels are sufficient until hatching to fulfill their functions. This is concomitant with immunochemistry data using an antibody recognizing both isoforms where during early development of medaka embryo all cells stained moderately for Egfr until late blastula. Then, at 24-somite stage, staining was detected only in the surface epithelia, aorta, intestinal epithelium and pronephric duct [11].

In platyfish, *egfra *is slightly expressed in embryos whereas *egfrb *was not detected [36]. Moreover, in zebrafish, addition of Egfr kinase inhibitors resulted in cardiovascular defects in the developing embryo [7]. In the mouse, knock-out experiments pointed to a complex role of Egfr during embryogenesis and early development; mutant mice are growth retarded and die at different postnatal stages depending on their genetic background. Surviving mice present a wide range of abnormalities (see [37] for a review).

The Egfr ligand transcripts for *tgfa *and *ereg *appear to be maternally deposited into the medaka egg as well but are not detected in the embryo after the mid-blastula transition [Additional file 8: Supplemental figures S10C and S10F]. This points to no important contribution of *tgfa*to later embryonic development. However, mutant mice for *Tgfa*show defects in hair follicles, eye development and decreased forebrain neural progenitor cell proliferation [38,39]. Zygotic transcription is observed for *egf*, *hbegf areg*, *epgn *and *btc *[Additional file 8: Supplemental figures S10B, S10D, S10E, S10G and S10H], with different expression patterns according to the developmental stage. From knock-out mice experiments, Hbegf is known to be important for normal heart development [40]. The only growth factor gene to exhibit a strong transcription in medaka embryos is *areg*, with increasing levels of transcripts past stage 30, when for instance the blood vessel system develops. In mammals, Areg was shown to be the most potent mitogen for vascular smooth muscle cells [41].

**Figure 4:**
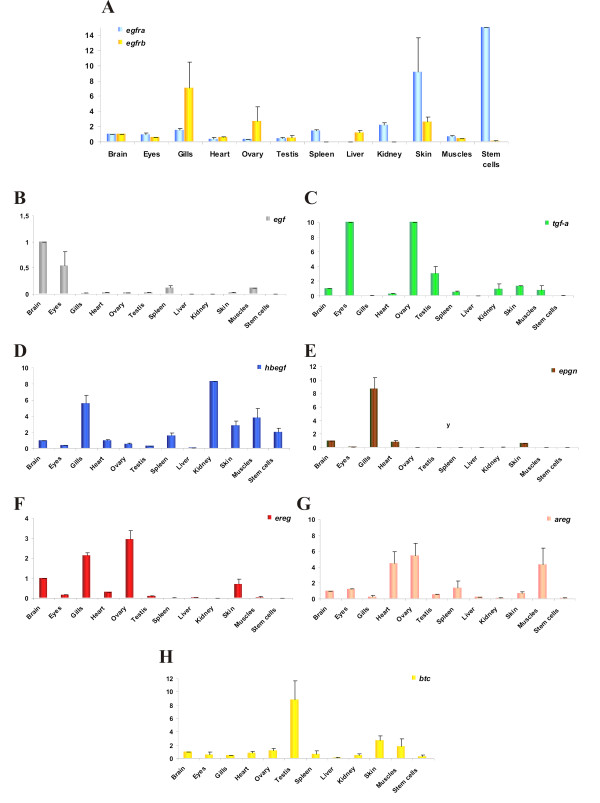
**Expression of Egf receptors and their ligands in medaka tissues and embryonic stem cells**. A) Expression of *egfra *and *egfrb*, B) *egf*, C) *tgfa*, D) *hbegf*, E)*epgn *, F)*ereg*, G)*areg *and H) *btc *in adult medaka tissues or organs, and in medaka stem cells. Values for each gene were normalized to expression levels of elongation factor 1 alpha 1 (*ef1a1*) using the 2-DDCT method [52]. Expression in brain was set as reference (value: 1); Data are presented as mean ± standard deviation of three independent quantitative real-time PCR experiments.

Medaka embryonic stem (ES) cells, which are blastula-derived cells, displayed large amounts of *egfra *mRNA, whereas *egfrb *was only weakly expressed (Figure 4A). Only one EGFR ligand is thoroughly expressed in the medaka ES cells, namely *hbegf *(Figure 4D). Low expression levels are observed for *btc *(Figure 4H) and *areg *(Figure 4G), but all the other ligands are not present in medaka ES cells.

In summary, the specific expression pattern of the seven medaka EGFR ligands during embryogenesis suggest different functions, although the possibility for signaling redundancy by autocrine, paracrine and/or juxtacrine modes of action remains (for a review see [42]).

#### Expression in adult tissues and organs

In adult medaka, both receptors are transcribed to a similar extent in brain, eye, heart, testis and muscles. However, *egfra *is expressed more strongly in spleen, kidney, and skin as well in stem cells, while *egfrb *is prominently expressed in gills, ovary and liver (Figure 4A). These results are consistent with the observations made in the platyfish [36]. These distinct expression profiles for *egfra *and *egfrb *may reflect different functions for the two *egfr *genes.

In medaka ovary, prominent expression of *egfrb *is accompanied with the presence of *tgfa, ereg *and *areg *(Figures 4C, F and 4G). In kidney, only *egfra *is transcribed, together with *hbegf *(Figure 4D). Both receptors are present in skin, *egfra *being the major transcript. Here, *hbegf *is mostly strongly expressed compared to other ligands but presence of *tgfa*, *areg *and *ereg *is also detectable to a lesser extent.

Taken together, the partly divergent expression patterns indicate that the two copies of the medaka *egfr *gene may have been preserved by subfunctionalization, a process where the ancestral gene functions are distributed among duplicated genes [14,43,44]. Moreover, the two receptors not only show dissimilar expression patterns but are co-expressed with different subsets of ligands. In general, *egfra *is co-expressed with *hbegf*, while *egfrb *is co-expressed with *tgfa*, *areg *and *ereg*.

### Functionality of the medaka Egfra-Egfr ligand system

The low conservation of ligand/receptor protein interface [Additional file 6] calls into question the usage of heterologous ligands for biochemical studies. In the few existing works on Egfr signaling within fish species, only recombinant heterologous ligands from mammals have been used to activate fish Egfrs [9,17,18]. In these studies, mammalian Egf, but also Tgfa, Hbegf and Btc were described to cross-react with the fish receptor. However, for the platyfish, only human EGF, but neither human TGFA nor mouse Egf was able to activate Egfra and induce a mitogenic effect [36]. As the Egf receptor ligand binding pocket is not well conserved between tetrapods and teleosts, the use of heterologous ligand to activate fish Egfr may differ and could hamper or event prevent the biological output. To analyze on the one hand the functionality of medaka Egfr and on the other hand its possible cross-specificity with human EGF, we performed stimulation experiments using either recombinant human EGF or a donor cell line expressing medaka ligands to stimulate a receptor cell line which over-expressed medaka *egfr*. We decided to examine only medaka Egfra activation, as this receptor is more related to human EGFR than Egfrb, thus rendering Egfra a more likely candidate for ligand cross-reactivity.

**Figure 5:**
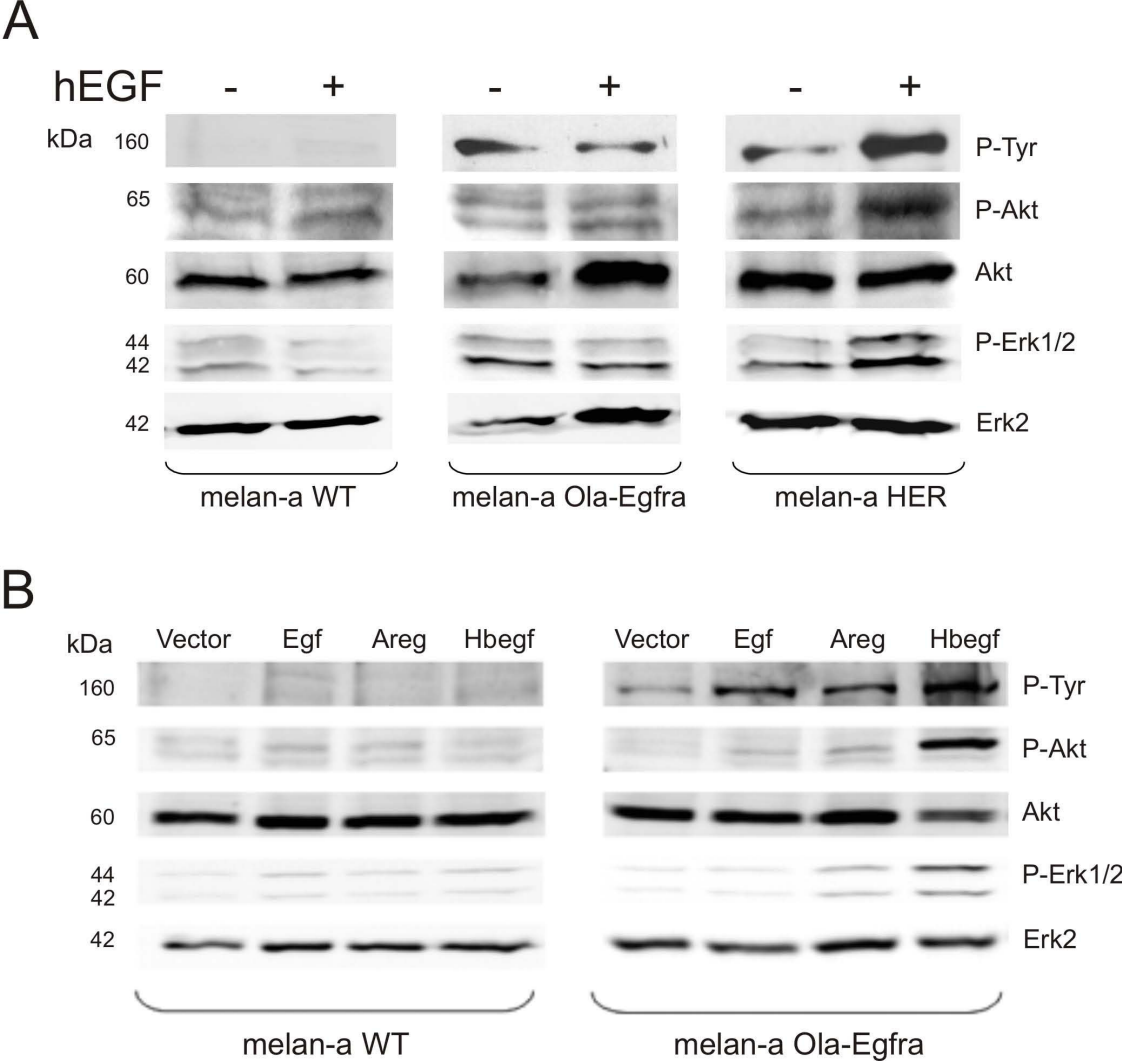
**Western blot analysis of medaka Egfra activation by human EGF and by medaka Egfr ligands**. A) Melan-a WT, melan-a Ola-*egfra *and melan-a *HER *cells were stimulated for 30 minutes with 100 ng/ml of recombinant human EGF at 37°C. P-Tyr: phospho-tyrosine antibody; P-Akt: phospho-Akt antibody; Akt: Akt antibody; P-Erk1/2: phospho-Erk1/2 antibody; Erk2: Erk2 antibody. B) Melan-a cells WT or melan-a Ola-*egfra *stimulated for 30 minutes at 37°C with overnight conditioned supernatant of 293-T cells expressing vector alone, medaka *egf*, medaka *areg *or medaka *hbegf*. P-Tyr: phospho-tyrosine antibody; P-Akt: phospho-Akt antibody; Akt: Akt antibody; P-Erk1/2: phospho Erk1/2; Erk2: Erk2 antibody. The anti-P-Tyr antibody recognizes a band of the size of Egfra, thus indicating activated receptor.

First, we applied recombinant hEGF on murine melanocyte wild-type cells (melan-a WT, lacking endogenous Egfr), melan-a cells that were stably transfected with medaka *egfra *(melan-a Ola-*egfra*) or melan-a cells stably transfected with human *EGFR *(melan-a *HER*). As shown by Western blot analysis, only melan-a *HER *cells displayed EGF-dependent phosphorylation of EGFR and the downstream signaling components Akt and Erk1/2 (Figure 5A). In case of melan-a Ola-*egfra *cells, hEGF treatment had no visible effect, thus speaking against cross-reactivity between hEGF and the medaka receptor (Figure 5A).

As Egfra did not respond to hEGF, we had to examine its functionality with species-specific ligands. As receptor cell line we used melan-a Ola-*egfra *cells and as donor cell line we transiently transfected human embryonic kidney (HEK) 293-T cells with expression vector constructs containing the cDNA of different medaka ligands (pCS2+-Ola-*egf*, pCS2+-Ola-*areg*, pCS2+-Ola-*hbegf*). Expression of the different constructs was verified by PCR [Additional file 9]. Wild-type melan-a cells and melan-a Ola-*egfra *cells were then stimulated for 30 minutes with conditioned supernatants from the transfected 293-T cells and activation of receptor and downstream signaling pathways was assessed by Western blot analysis. As expected, melan-a WT cells did not display any activation of Egf receptor and its downstream signaling pathways in response to any of the conditioned supernatants (Figure 5B). In contrast, melan-a Ola-*egfra *could be stimulated with Ola-Egf, Ola-Areg or Ola-Hbegf conditioned supernatants as demonstrated by anti phospho-Tyr (P-Tyr) staining of a protein with the size of medaka Egfra, that was only visible in cells expressing this receptor (Figure 5B). Ola-Hbegf conditioned supernatant showed the strongest effect on receptor-, Akt- and Erk1/2 phosphorylation, whereas the response was much weaker with Ola-Egf and Ola-Areg conditioned supernatants. However, a clear receptor phosphorylation was always and repeatedly observed in independent experiments. An explanation for the weak activation of downstream pathways by medaka Egf and Areg may indicate a better reactivity of medaka Egfra with medaka Hbegf when compared to other ligands, corroborating the expression patterns in adult organs data where medaka *egfra *was found to be preferentially expressed together with *hbegf*. Differences in receptor- or Erk1/2 turnover in response to different stimuli or, alternatively, different ligand half-lives in solution are other possible explanations for the observed differences.

Altogether, whereas recombinant hEGF did not trigger any response, all three medaka ligands led to activation of medaka Egfra or downstream pathways, indicating receptor functionality.

## Conclusions

A detailed examination of the medaka Egfr/Egfr ligand system, considering both functional and evolutionary aspects, revealed that the medaka genome contains two Egfr gene copies, namely *egfra *and *egfrb*, but only one copy of each of the seven Egfr ligand genes. Including conserved synteny data from various vertebrate genomes, we found that most likely all present vertebrate Egfr ligands date back to a single Egfr ligand gene, which gave rise to the Egfr ligand repertoire found in extant teleost genomes.

All investigated fish Egf receptor genes occur in two copies that have been maintained in the course of teleost evolution. This may be due to subfunctionalization and specialization of the two receptor proteins. This theory is supported by the differential expression of both genes in medaka embryo and adult tissues, together with the expression of a distinct subset of ligand genes.

Finally, we assessed medaka Egf, Areg and Hbegf functionality, demonstrating that all of them could trigger medaka Egfra receptor activation. This was not accomplished using human EGF. Consequently, we strongly support the use of homologous ligands as prerequisite in future functional biochemical studies.

## Methods

### Database surveys

All the species used in this study are listed in [Additional file 10: Supplemental table S2]. Using human and fish sequences as queries, searches for Egfr and Egfr ligand sequences was performed in databases accessible with the Basic Local Alignment Search Tool (BLAST) on the servers of the National Center for Biotechnology Information (NCBI), the Ensembl Database http://www.ensembl.org/. A list of the gene accession numbers is given in [Additional file 10: Supplemental table S3].

The sequences of *X*. *maculatus tgfα*, *hbegf*, *btc*, *ereg *and *epgn *were obtained blasting the published medaka amino-acid sequences against the *Xiphophorus *ESTs database (R. B. Walter, unpublished data; sequences available under accession numbers GU144290 to GU144294). In the search of Egf receptor ligands, only the mature Egf motif was used to perform the BLAST searches since this is the only conserved domain [Additional file 2].

### Phylogenetic tree reconstruction

The nucleotide sequences of the Egf motif of the Egfr ligands from tetrapod and teleost species were retrieved from Ensembl and NCBI databases, loaded into BioEdit [45], translated into amino acid sequences and aligned with CLUSTALW [46] as implemented in BioEdit. Maximum likelihood phylogenies based on the nucleotide sequences were inferred with PHYML Online [47] using the GTR+I+R model and 500 bootstrap replications. A bootstrap consensus tree from this analysis was then generated and drawn with MEGA4 [48]. Neighbor Joining bootstrap values of 10,000 replications were also obtained from MEGA4.

### Synteny analyses

Synteny analyses of *Egf*, *Tgfa*, *Hbegf *and the ligand cluster were performed using information from the genome browsers from human, mouse, chicken, zebrafish, *Tetraodon *and medaka from the Ensembl database http://www.ensembl.org/.

The Synteny Database [20,21] was used for large-scale synteny analyses. Generally, 20 Mb around the human EGFR ligand genes were taken into account. For the analysis of paralogous relationships within the human genome, amphioxus was chosen as outgroup genome. The *Ciona *genome as outgroup gave similar results. The results were then compared to previously published synteny regions [19,22] to infer protochromosome connections.

### Fishes and fish cell lines

All animal studies have been approved by the author's Institutional Review Board (Animal Welfare Officer of the University of Würzburg). Fishes were kept under standard conditions in the aquarium facility of the Biozentrum at the University of Würzburg. The medaka *Carbio *strain (outbred strain, mixed genetic background of southern medaka) was used in this study. The origin of the medaka stem cell and fibroblast cell lines has previously been published previously [49]. Embryonic developmental stages are described as defined by Iwamatsu [50].

### RNA extraction, first strand DNA synthesis and real-time PCR analysis

RNA extraction from different medaka tissues and cell lines was done using Total RNA Isolation Reagent (ABgene, Epsom, UK) as recommended by the manufacturer. For analysis of gene expression, standard PCR for control of gene expression or real-time PCR for the expression pattern analyses was done. For the embryonic medaka stages study, a pool of 10 embryos per stage was prepared and the early morula stage, stage 8, was chosen as expression reference. Given that Egfr signaling is observed in the CNS, a feature conserved from invertebrates to vertebrates, we took medaka adult brain as a reference organ for the adult tissues. cDNA was prepared from total RNA from a pool of 6 adult fish brains, eyes, gills, hearts, ovaries, testes, spleens, kidneys, livers, skins, fins, muscles, as well as medaka stem cells line using the RevertAid kit with random hexamer primers (Fermentas, Burlington, Canada). PCR primers were designed using Primer3 software [51]. A list of the different primer combinations is given in [Additional file 10: Supplemental table S4]. For the different embryonic stages, adults organs and the stem cell line each PCR was carried out in duplicate and was repeated 3 times independently in a 25 μl volume using a SYBR green containing master mix for 4 minutes at 95°C for initial denaturation followed by 40 cycles of 95°C for 30 seconds and 60°C for 60 seconds in the iCycler IQ (Bio-Rad, Hercules, CA). All PCR products were confirmed by sequencing using the CEQ DTCS dye terminator cycle sequencing kit and run on a CEQ 2000XL DNA sequencing system (Beckmann Coulter, Krefeld, Germany). Values for each gene were normalized to expression levels of elongation factor 1 alpha 1 (*ef1a1*) using the 2-DDCT method [52].

### ConSurf analysis

ConSurf http://consurf.tau.ac.il/[35], an automated web based server, was used to project the site conservation grades between tetrapod and teleost Egfr onto the molecular surface of the human EGF receptor protein in complex with TGFA [33]. In this study, the rate of evolution at each site was calculated using the empirical Bayesian paradigm that was shown to significantly improve the accuracy of conservation scores estimations over the Maximum Likelihood method, in particular when a small number of sequences was used for the calculations [53].

Using this tool, we could display the patches of low or highly conserved residues that are important for ligand binding. Confidence intervals were generated and high and low values of each interval were assigned color grades according to the 1-9 coloring scheme. If the interval in a specific position spans 4 or more color grades, the score is considered as unreliable and the position is colored light yellow in the graph. The positions indicated in the figure refer to the positions of the human mature EGFR protein.

### Cloning and sequencing of medaka *egfra*, *egf*, *areg *and *hbegf*

To isolate medaka *egfra*, *egf*, *areg *and *hbegf*, we searched the medaka genome database http://www.ensembl.org/Oryzias_latipes/index.html by tBLASTN and BLASTn comparison [54]. We identified the corresponding genes on chromosome 17 (*egfra*), ultracontig115 (*egf*), chromosome 9 (*areg*), and chromosome 10 (*hbegf*).

The deduced mRNAs of the different genes were predicted by using GeneScan analysis [55]. The coding region of medaka *egfra*, *egf*, *areg *and *hbegf *were amplified from cDNA prepared from pooled medaka tissues at standard PCR conditions using Triple Master Taq polymerase proof-reading Taq polymerase (Eppendorf, Westbury, NY) (primer sequences available on request). The PCR products were inserted directly by TA cloning into the pCR2.1 vector (TA Cloning Kit, Invitrogen), and individual clones were sequenced using CEQ DCTS dye terminator cycle sequencing kit and run on a CEQ 2000XL DNA sequencing system (Beckman-Coulter).

### Expression constructs for Medaka *egfra*, *egf*, *areg *and *hbegf*

*Xba *I and *Sna*BI restriction sites were inserted at the 5' and the 3' termini of medaka *egfra*, respectively, in order to ligate the gene into the *Xba*I and *Sna*BI digested PCS2+ expression vector to create the construct pCS2+-Ola-*egfra*.

To generate the different pCS2+ expression constructs for medaka *egf*, *areg *and *hbegf*, *Eco*RI and *Xba*I restriction sites were inserted at the 5' and 3' termini of medaka *egf*, *Eco*RI and *Xho*I were inserted at the 5' and 3' termini of medaka *areg *and *Cla*I and *Xba*I were inserted at the 5' and 3' termini of medaka *hbegf *to insert them in the corresponding digested pCS2+ vector. Expression of medaka *egfra*, *egf*, *areg *and *hbegf *by the different cell lines was assessed by RT-PCR [Additional file 9].

### Cell culture, transfections and stimulation assays

Mouse wild-type melanocytes (melan-a WT) [56], lacking endogenous *Egfr *expression [57,58], were cultured in DMEM, 10% FCS in the presence of cholera toxin (12 nmol/L), and TPA (200 nmol/L). Melan-a WT cells were then stably transfected with the pCS2+-Ola-*egfra *construct using Fugene transfection reagent (Roche). HEK 293T cells (human embryonic kidney fibroblasts with SV40 T-antigen) were grown in DMEM (Invitrogen, Karlsruhe, Germany) supplemented with 10% FCS, 1% glutamine and antibiotics. Expression constructs pCS2+-Ola-*egf*, pCS2+-Ola-*areg *and pCS2+-Ola-*hbegf *were transiently transfected into HEK 293T cells by the calcium-phosphate method [59]. Expression of medaka *egfra *in melan-a cells and medaka *egf*, *areg *and *hbegf *RNA in 293T cells was checked by reverse-transcription PCR analysis.

For the stimulation assays, cells were incubated either with human EGF (100 ng/ml) (Tebu-bio, Offenbach, Germany) or with the 293T cells overnight conditioned supernatants for 30 minutes at 37°C.

### Cell lysis and Western blotting

Cells were trypsinized, rinsed twice with PBS and lyzed in 50 mmol/L HEPES (pH 7.5), 150 mmol/L NaCl, 1.5 mmol/L MgCl_2_, 1 mmol/L EGTA, 10% glycerol, 1% Triton X-100, 10 μg/mL aprotinin, 10 μg/mL leupeptin, 200 μmol/L Na_3_VO_4_, 1 mmol/L phenylmethylsulfonyl fluoride (PMSF), and 100 mmol/L NaF. 50 μg of protein lysate was separated by SDS-PAGE and analyzed by Western blotting onto nitrocellulose. Membranes were blocked for 60 minutes with TBS (10 mmol/L Tris-HCl (pH 7.9) and 150 mmol/L NaCl), 0.1% Tween-20, and 5% bovine serum albumin (BSA) and were incubated overnight at 4°C with the first antibody. Monoclonal antiphosphotyrosine P-Tyr (PY20) was from BD Biosciences (San Jose, CA). Phospho-ERK 1/2 (Thr202/Tyr204) and rabbit polyclonal Akt antibodies were purchased from Cell Signaling Technology (Danvers, MA). Rabbit polyclonal anti-phospho Akt (Ser473) was obtained from New England Biolabs (Ipswich, MA) and rabbit polyclonal anti-Erk2 (c-14) from Santa Cruz Biotechnology (Santa Cruz, CA). The secondary antibodies were conjugated with horseradish peroxidase and were directed against mouse (Pierce, Rockford, IL) or rabbit (Bio-Rad).

## Authors' contributions

JAGCL performed the computational search for tetrapod and teleost *Egfr *and Egfr ligand genes, the synteny analyses, the study of Egfr subdomains and Egfr extracellular part evolutionary conservation, the expression analyses, the cloning and sequencing of medaka *egfra*, *areg*, *egf *and *hbegf*, the functional experimentations and wrote the manuscript. IB did the phylogenetic analysis, the large-scale synteny examination, the study of vertebrate EGFR ligand evolution and wrote the manuscript. RBW generated and provided the *Xiphophorus maculatus *sequences for *tgfα*, *hbegf*, *btc*, *ereg *and *epgn*. MS produced and supplied the fish material. SM and MS both designed the study and helped draft the manuscript. All authors read and approved the final manuscript.

## Supplementary Material

Additional file 1 **Supplemental figure S1. Structure and amino acid sequence alignment for tetrapod and teleost Egfr**. A) Overall Egfr structure comprising the amino-terminus (NH2), the extracellular domain (ECD), the transmembrane domain (TM), the intracellular juxtamembrane domaine (JM), the intracellular tyrosine kinase domaine (TK) and the carboxy-terminus (COOH). B) Alignment was generated in ClustalX. The color bars indicate the different subdomains of the Egfr protein: subdomain I in green, subdomain II in blue, subdomain III in magenta, subdomain IV in orange, transmembrane domain in yellow, intracellular juxtamembrane in grey and tyrosine kinase in red. Click here for file

Additional file 2 **Supplemental figure S2. Amino acid sequence alignment for tetrapod and teleost Egfr ligands**. Alignment was generated in ClustalX [46]. Only the conserved Egf motif DNA sequences were used for the analysis. Click here for file

Additional file 3 **Supplemental figure S3. Phylogeny of EGFR ligand genes**. Original Maximum Likelihood tree based on the 138 bp of the Egf domain using the GTR+G+I substitution model. Bootstrap values for Maximum Likelihood and Neighbor Joining methods are shown. The tree was rooted on the branch leading to *Egf *sequences. Only bootstrap values above 50% are shown. Monophyly is supported for *Egf*, *Hbegf*, *Tgfa *and *Ereg *genes, but many other nodes remain poorly supported. Dre, *Danio rerio*; Gac, *Gasterosteus aculeatus*; Gga, *Gallus gallus*; Hsa, *Homo sapiens*; Mmu, *Mus musculus*; Ola (red), *Oryzias latipes*; Ssa, *Salmo salar*; Tni, *Tetraodon nigroviridis*; Tru, *Takifugu rubripes*; Xma, *Xiphophorus maculatus *[Additional file 10: Supplemental table S3]. Click here for file

Additional file 4 **Supplemental figures S4 to S7**. Synteny Database [21] dot plots showing orthologous genes (red crosses) from human *EGF *(S4), *TGFA*(S5), *HBEGF*(S6) and the ligand cluster (S7) regions. Click here for file

Additional file 5 **Supplemental figure S8. Structure and amino acid sequences alignment for human and medaka Egfr**. A) Overall Egfr structure comprising the amino-terminus (NH2), the extracellular domain (ECD), the transmembrane domain (TM), the intracellular juxtamembrane domaine (JM), the intracellular tyrosine kinase domaine (TK) and the carboxy-terminus (COOH). B) Alignment was generated in ClustalX. The color bars indicate the different subdomains of the Egfr protein: subdomain I in green, subdomain II in blue, subdomain III in magenta, subdomain IV in orange, transmembrane domain in yellow, intracellular juxtamembrane in grey and tyrosine kinase in red. Major phosphorylation sites in the carboxy terminal tail are indicated by asterisks (*). Click here for file

Additional file 6 **Supplemental table S1. Amino acid similarity between tetrapod and teleost Egf receptor extracellular subdomains**. Similarity percentages between human (Hsa), mouse (Mmu), chicken (Gga), medaka (Ola), platyfish (Xma), zebrafish (Dre), green-spotted pufferfish (Tni), fugu (Tru) and three-spined stickleback (Gac) Egfr extracellular subdomains I, II, III and IV. Click here for file

Additional file 7 **Supplemental figure S9. ConSurf evolutionary conservation analysis of the Egfr ligand binding pocket residues between tetrapods and teleosts**. A) Overall strucure of extracellular subdomains I and III of Egfr in complex with Tgfa, 3 interface sites are outlined. B) View of site 1 interface. C) View of sites 2 and 3 interfaces. Non-conserved residues are colored in turquoise whereas conserved residues are coloured in pink and maroon. Yellow color indicates amino acids for which data were not sufficient to calculate reliable conservation values. D) Table displaying the residues of the ligand binding pocket in human EGFR and medaka Egfra and Egfrb. Bold font indicates amino acid changes in either medaka Egfra or Egfrb compared to human EGFR. Amino acid substitutions that also involve an important change in the amino acid physicochemical properties are quoted by a (R) for radical amino acid substitution. Some of the residues shown to directly interact with Egf [34] or with Tgfa [33] (D) such as Tyr89 (site 1), Asp355 (site 2) or Leu17 and Glu384 (site 3) are well conserved. However, many residues directly interacting with the ligand or surrounding the binding site are not evolutionary conserved (B and C). In some cases amino acid substitution leads to an important shift in the amino acid property, e.g., positively vs. negatively charged [60]. For example, aromatic Phe20 of mammals is replaced in teleosts by basic Lys or Arg, nucleophilic Thr or acidic Glu. Gln47 in mammals is replaced in teleosts by acidic Asp or Glu, neutral Ala or hydrophobic Leu. Similar radical amino acid substitutions are also observed for Ala103 and Lys105. Regarding site 2 (C), ligand-interacting Asp355 is well conserved between tetrapods and teleosts, but radical amino acid substitutions occur for other interacting residues, like the aromatic Phe357 which is replaced in teleost Egfrb by a basic His. Here again, many amino acids located at the interface show no conservation, like for Pro349, Arg353, Ser356 and His359. At site 3, even more radical amino acid substitutions were observed, *e.g*. for His409, Phe412, Val417, and Ile438. Click here for file

Additional file 8 **Supplemental figure S10. Expression of Egf receptors and their ligands in medaka embryo**. A) Expression of *egfra*, *egfrb*, B) *egf*, C) *tgfa*, D) *hbegf*, E)*epgn *, F)*ereg*, G)*areg *and H) *btc *in medaka embryo stages 8, 24, 27-28, 30, 37-38 and 39. Values for each gene were normalized to expression levels of elongation factor 1 alpha 1 (*ef1a1*) using the 2-DDCT method [52]. Data are presented as mean ± standard deviation. Expression at stage 8 was set to one fold as a reference; data are average values of three independent quantitative real-time PCR experiments. Click here for file

Additional file 9 **Supplemental figure S11. PCR analysis of medaka *egfra*, *egf*, *areg *and *hbegf*expression in melan-a and 293T cells**. A) Expression of medaka *egfra *in melan-a WT and melan-a Ola-*egfra*cells. B) Expression of medaka *egf*, *areg *and *hbegf *in 293T cells transiently transfected with the expression vector alone (vector), medaka *egf *(*egf*), medaka *areg *(*areg*) or medaka *hbegf *(*hbegf*). Click here for file

Additional file 10 **Supplemental tables S2 to S4**. S2) List of all species used in this analysis. S3) Accession numbers of all genes used in this analysis. S4) List of primers used for quantitative real-time PCR analyses. Click here for file
