# Supplementary figures and images for: Lineage-specific co-evolution of the Egf receptor/ligand signaling system
Source: BMC Evol Biol. 2010 Jan 27;10:27. doi: 10.1186/1471-2148-10-27 (PMC2834686; doi:10.1186/1471-2148-10-27)

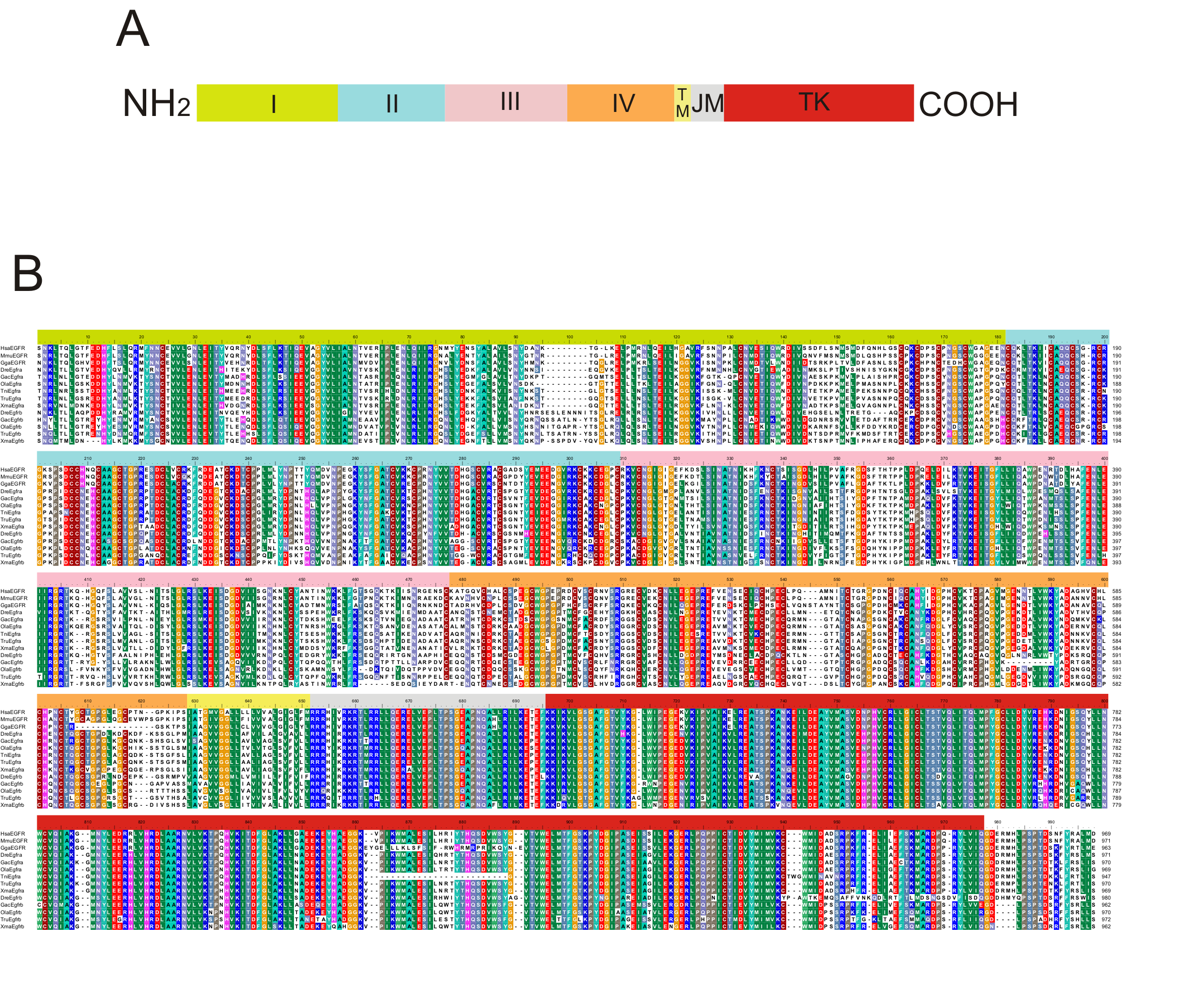

Supplement: Additional file 1 — Supplemental figure S1. Structure and amino acid sequence alignment for tetrapod and teleost Egfr. A) Overall Egfr structure comprising the amino-terminus (NH2), the extracellular domain (ECD), the transmembrane domain (TM), the intracellular juxtamembrane domaine (JM), the intracellular tyrosine kinase domaine (TK) and the carboxy-terminus (COOH). B) Alignment was generated in ClustalX. The color bars indicate the different subdomains of the Egfr protein: subdomain I in green, subdomain II in blue, subdomain III in magenta, subdomain IV in orange, transmembrane domain in yellow, intracellular juxtamembrane in grey and tyrosine kinase in red. [file 1471-2148-10-27-S1.PNG]

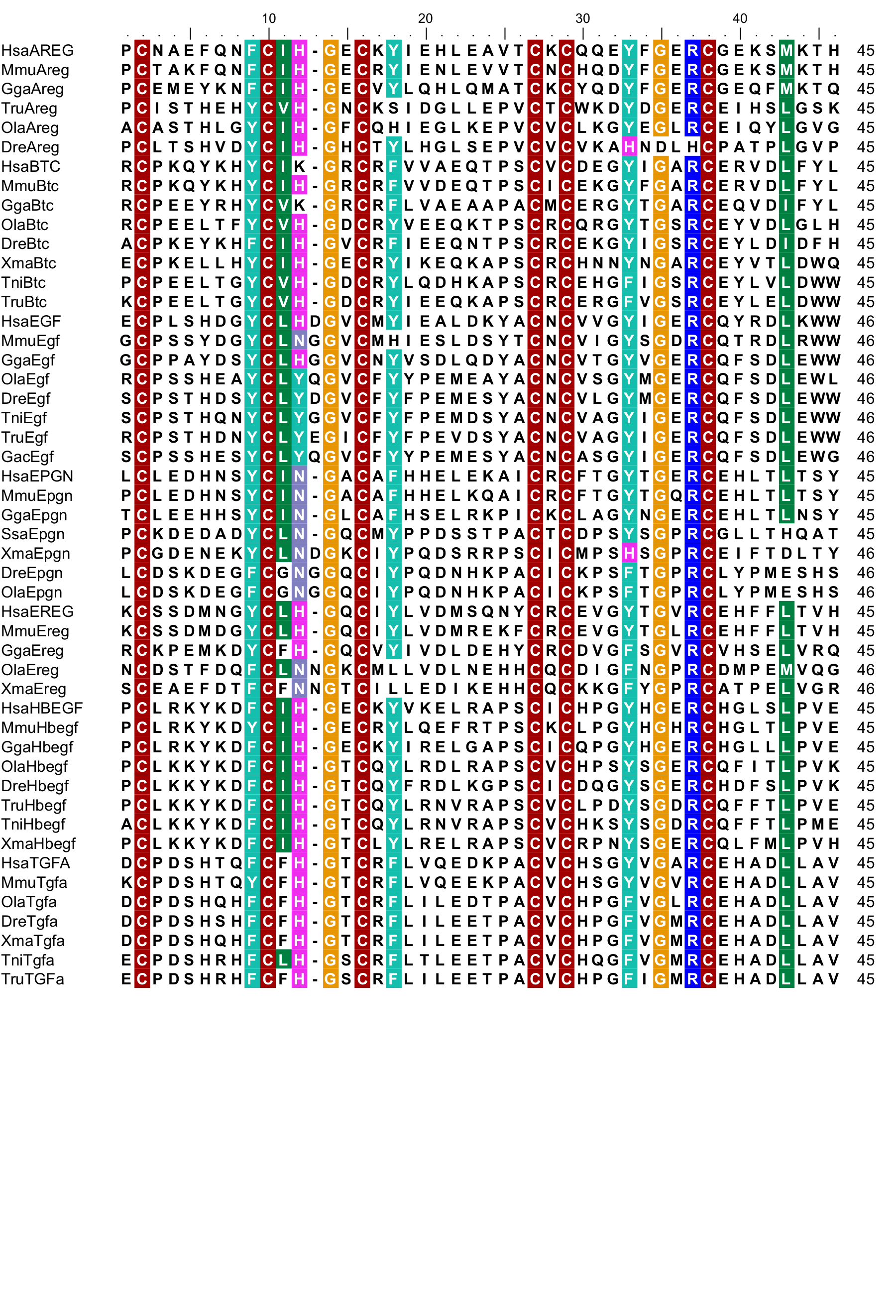

Supplement: Additional file 2 — Supplemental figure S2. Amino acid sequence alignment for tetrapod and teleost Egfr ligands. Alignment was generated in ClustalX [46]. Only the conserved Egf motif DNA sequences were used for the analysis. [file 1471-2148-10-27-S2.PNG]

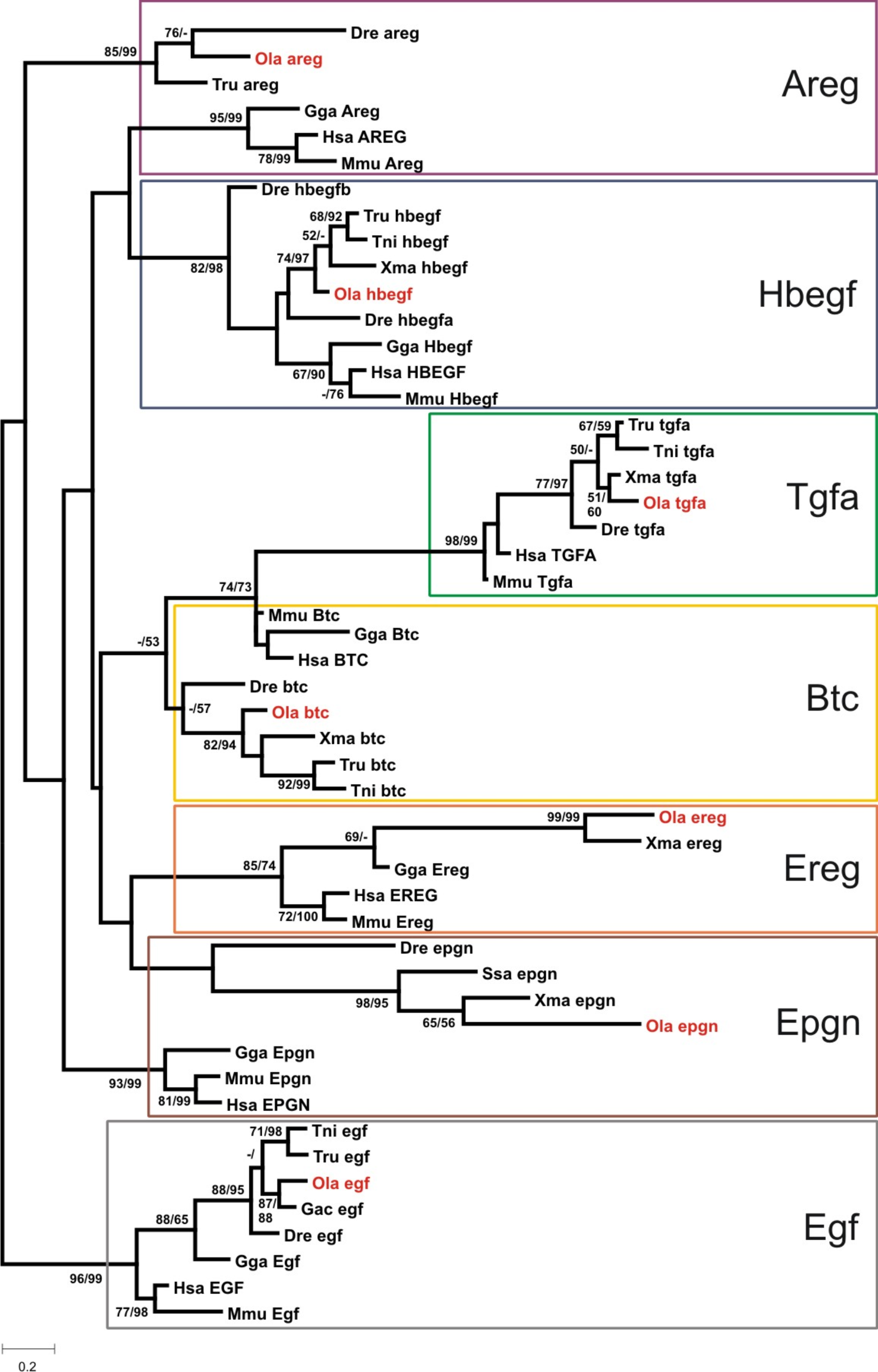

Supplement: Additional file 3 — Supplemental figure S3. Phylogeny of EGFR ligand genes. Original Maximum Likelihood tree based on the 138 bp of the Egf domain using the GTR+G+I substitution model. Bootstrap values for Maximum Likelihood and Neighbor Joining methods are shown. The tree was rooted on the branch leading to Egf sequences. Only bootstrap values above 50% are shown. Monophyly is supported for Egf, Hbegf, Tgfa and Ereg genes, but many other nodes remain poorly supported. Dre, Danio rerio; Gac, Gasterosteus aculeatus; Gga, Gallus gallus; Hsa, Homo sapiens; Mmu, Mus musculus; Ola (red), Oryzias latipes; Ssa, Salmo salar; Tni, Tetraodon nigroviridis; Tru, Takifugu rubripes; Xma, Xiphophorus maculatus [Additional file 10: Supplemental table S3]. [file 1471-2148-10-27-S3.PNG]

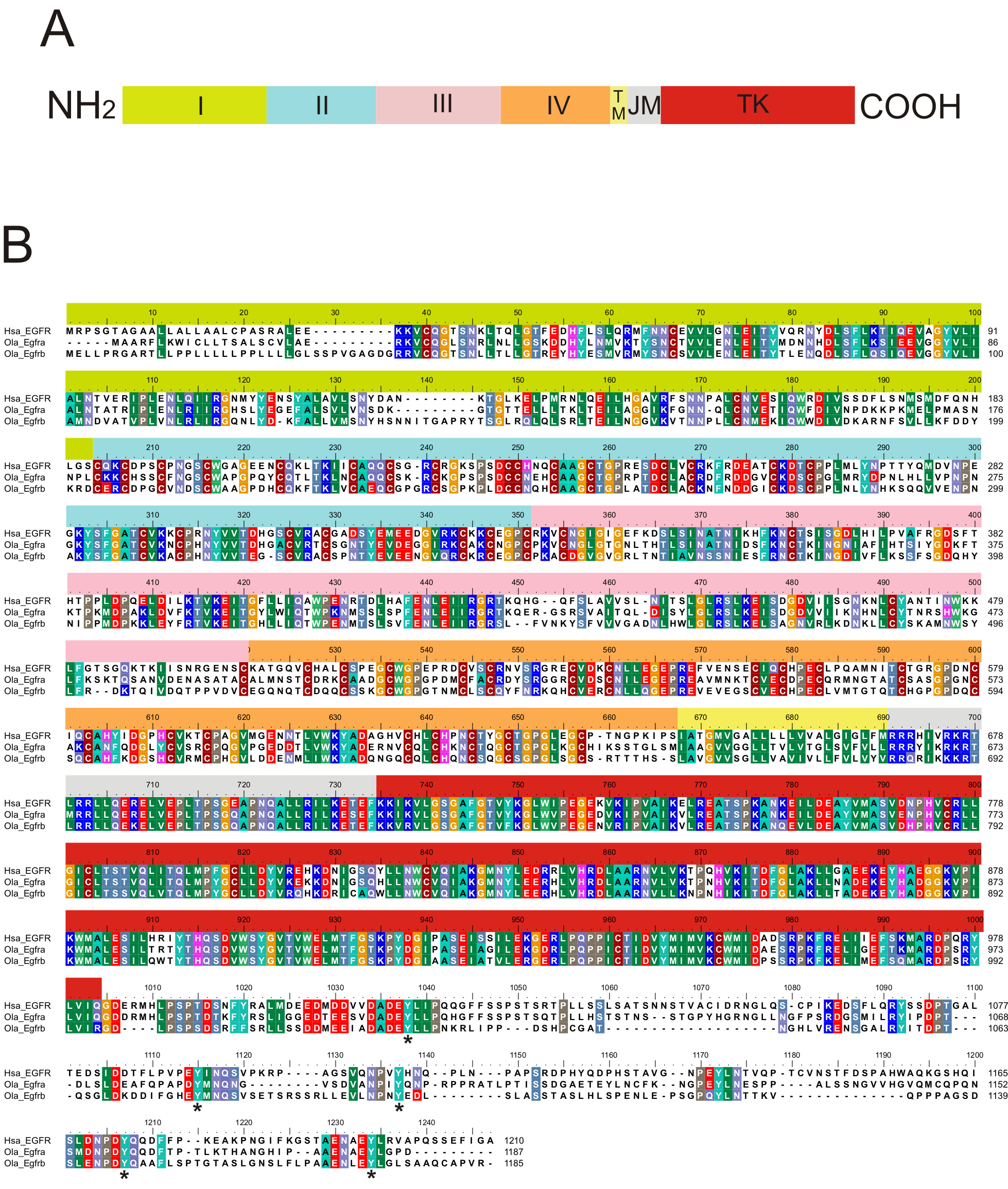

Supplement: Additional file 5 — Supplemental figure S8. Structure and amino acid sequences alignment for human and medaka Egfr. A) Overall Egfr structure comprising the amino-terminus (NH2), the extracellular domain (ECD), the transmembrane domain (TM), the intracellular juxtamembrane domaine (JM), the intracellular tyrosine kinase domaine (TK) and the carboxy-terminus (COOH). B) Alignment was generated in ClustalX. The color bars indicate the different subdomains of the Egfr protein: subdomain I in green, subdomain II in blue, subdomain III in magenta, subdomain IV in orange, transmembrane domain in yellow, intracellular juxtamembrane in grey and tyrosine kinase in red. Major phosphorylation sites in the carboxy terminal tail are indicated by asterisks (*). [file 1471-2148-10-27-S5.PNG]

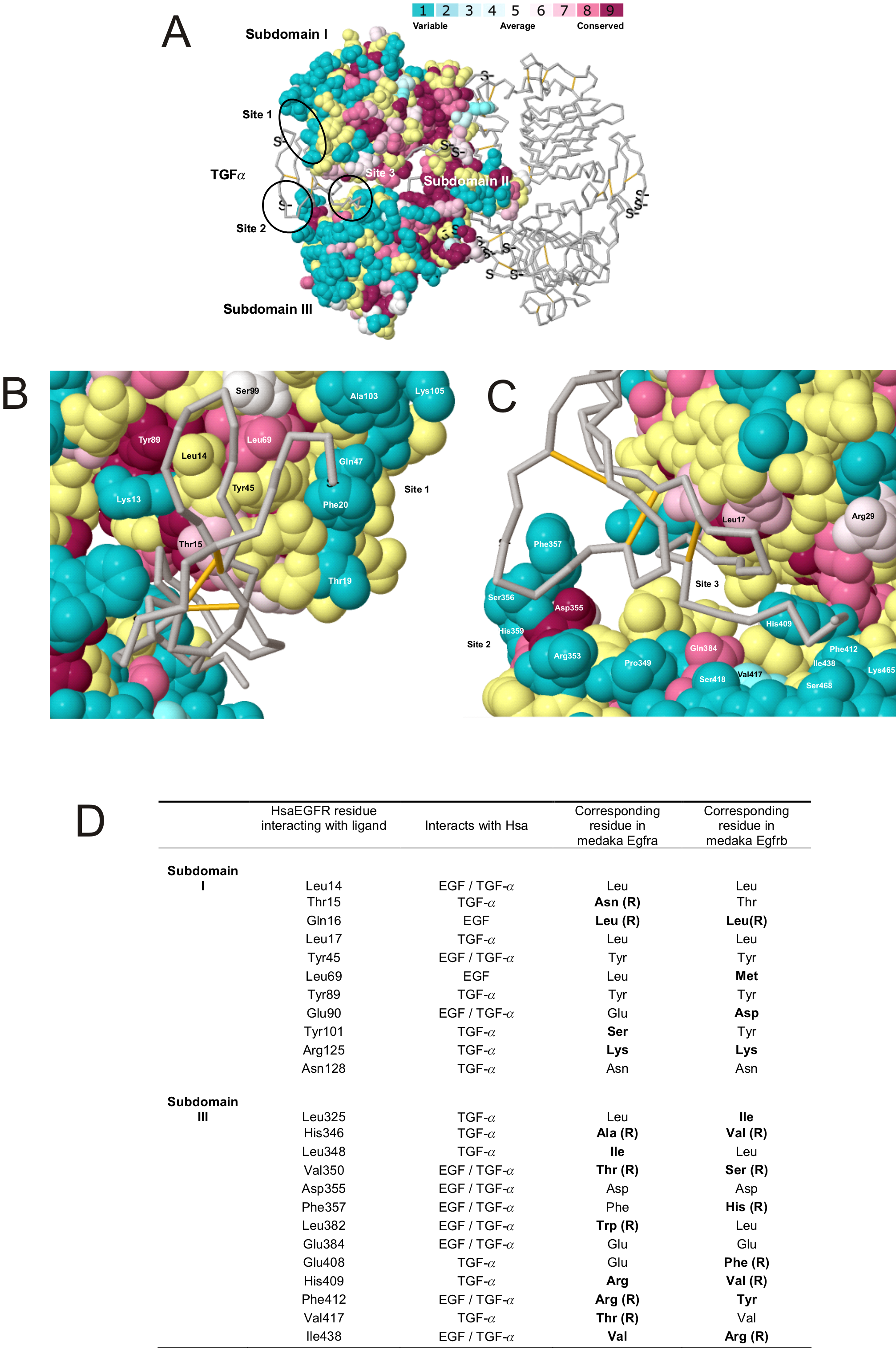

Supplement: Additional file 7 — Supplemental figure S9. ConSurf evolutionary conservation analysis of the Egfr ligand binding pocket residues between tetrapods and teleosts. A) Overall strucure of extracellular subdomains I and III of Egfr in complex with Tgfa, 3 interface sites are outlined. B) View of site 1 interface. C) View of sites 2 and 3 interfaces. Non-conserved residues are colored in turquoise whereas conserved residues are coloured in pink and maroon. Yellow color indicates amino acids for which data were not sufficient to calculate reliable conservation values. D) Table displaying the residues of the ligand binding pocket in human EGFR and medaka Egfra and Egfrb. Bold font indicates amino acid changes in either medaka Egfra or Egfrb compared to human EGFR. Amino acid substitutions that also involve an important change in the amino acid physicochemical properties are quoted by a (R) for radical amino acid substitution. Some of the residues shown to directly interact with Egf [34] or with Tgfa [33] (D) such as Tyr89 (site 1), Asp355 (site 2) or Leu17 and Glu384 (site 3) are well conserved. However, many residues directly interacting with the ligand or surrounding the binding site are not evolutionary conserved (B and C). In some cases amino acid substitution leads to an important shift in the amino acid property, e.g., positively vs. negatively charged [60]. For example, aromatic Phe20 of mammals is replaced in teleosts by basic Lys or Arg, nucleophilic Thr or acidic Glu. Gln47 in mammals is replaced in teleosts by acidic Asp or Glu, neutral Ala or hydrophobic Leu. Similar radical amino acid substitutions are also observed for Ala103 and Lys105. Regarding site 2 (C), ligand-interacting Asp355 is well conserved between tetrapods and teleosts, but radical amino acid substitutions occur for other interacting residues, like the aromatic Phe357 which is replaced in teleost Egfrb by a basic His. Here again, many amino acids located at the interface show no conservation, like for Pro349, Arg35 [file 1471-2148-10-27-S7.PNG]

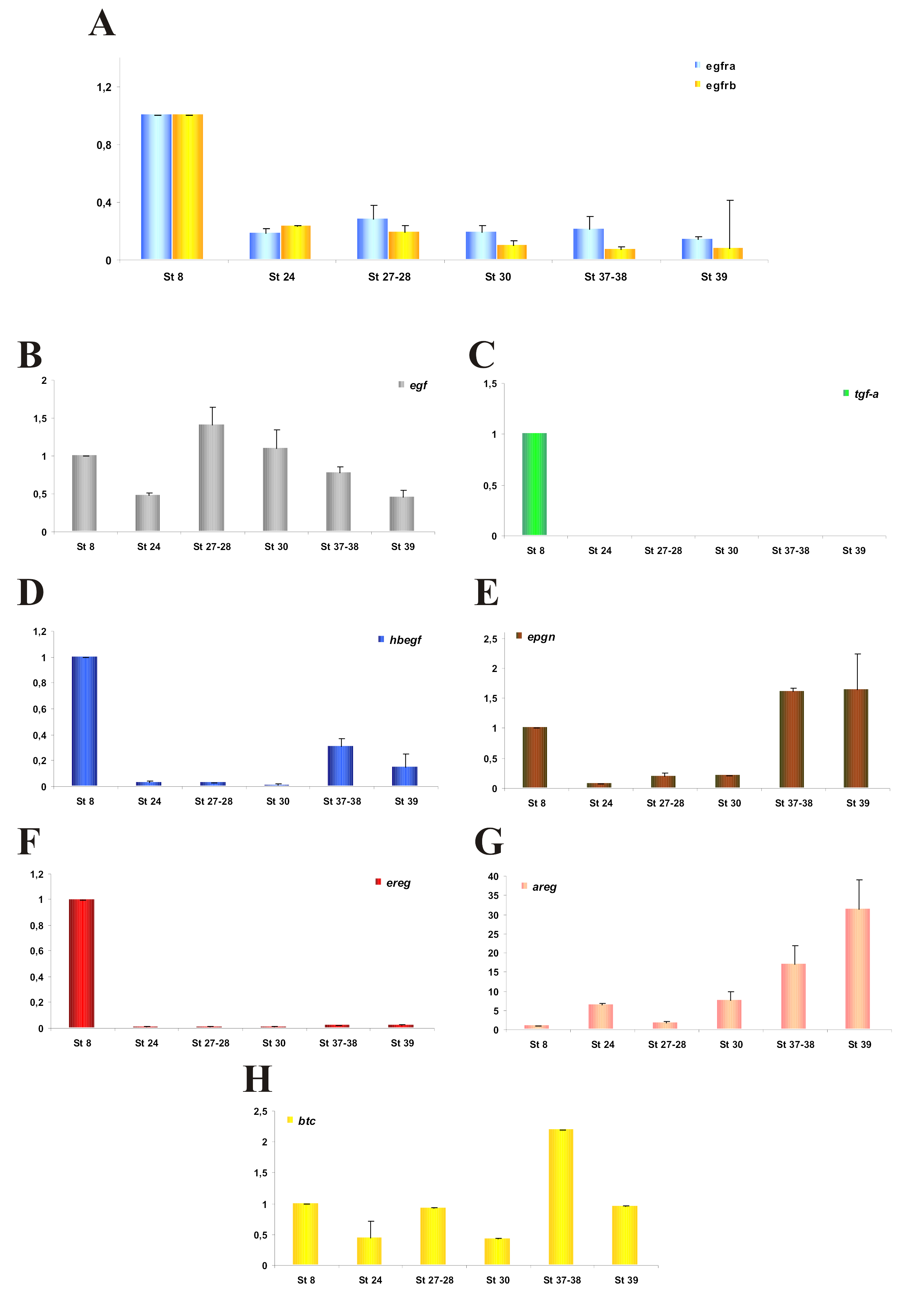

Supplement: Additional file 8 — Supplemental figure S10. Expression of Egf receptors and their ligands in medaka embryo. A) Expression of egfra, egfrb, B) egf, C) tgfa, D) hbegf, E)epgn , F)ereg, G)areg and H) btc in medaka embryo stages 8, 24, 27-28, 30, 37-38 and 39. Values for each gene were normalized to expression levels of elongation factor 1 alpha 1 (ef1a1) using the 2-DDCT method [52]. Data are presented as mean ± standard deviation. Expression at stage 8 was set to one fold as a reference; data are average values of three independent quantitative real-time PCR experiments. [file 1471-2148-10-27-S8.PNG]

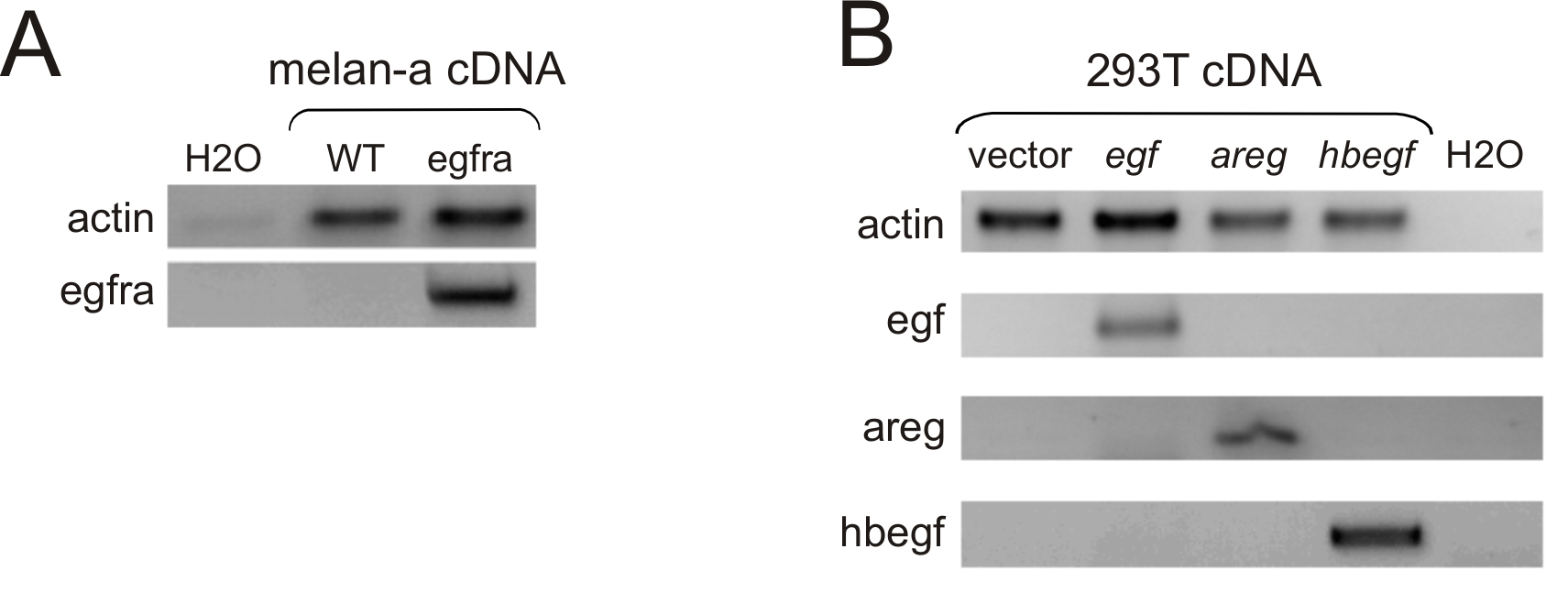

Supplement: Additional file 9 — Supplemental figure S11. PCR analysis of medaka egfra, egf, areg and hbegfexpression in melan-a and 293T cells. A) Expression of medaka egfra in melan-a WT and melan-a Ola-egfracells. B) Expression of medaka egf, areg and hbegf in 293T cells transiently transfected with the expression vector alone (vector), medaka egf (egf), medaka areg (areg) or medaka hbegf (hbegf). [file 1471-2148-10-27-S9.PNG]
