# Supplementary material for: Lineage-specific co-evolution of the Egf receptor/ligand signaling system
Source: BMC Evol Biol. 2010 Jan 27;10:27. doi: 10.1186/1471-2148-10-27 (PMC2834686; doi:10.1186/1471-2148-10-27)
Supplement: Additional file 4 — Supplemental figures S4 to S7. Synteny Database [21] dot plots showing orthologous genes (red crosses) from human EGF (S4), TGFA(S5), HBEGF(S6) and the ligand cluster (S7) regions. [file 1471-2148-10-27-S4.PDF]

S4

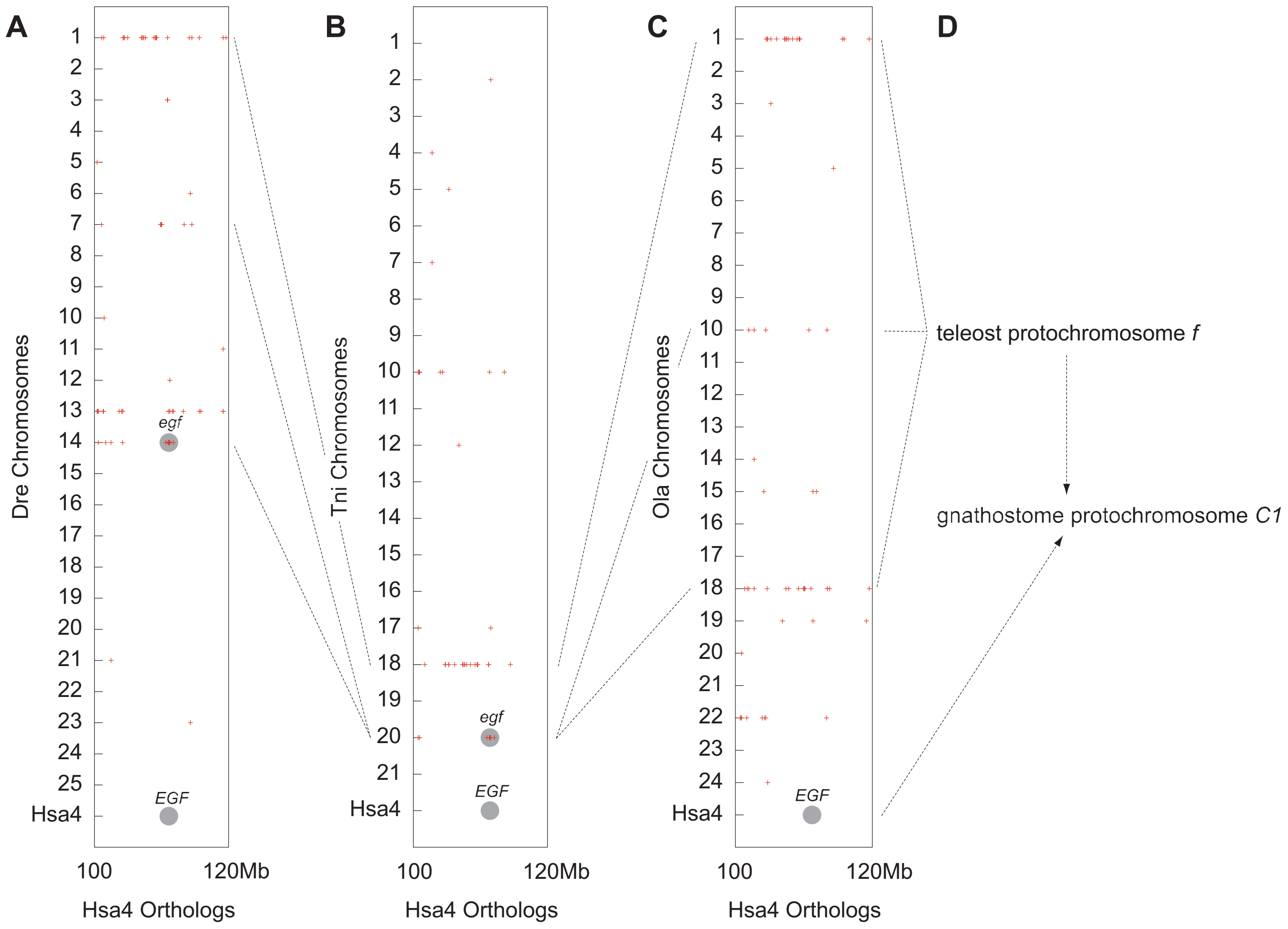

S4) Human EGF region on Hsa4 in the genomes of A) zebrafish, B) Tetraodon and C) medaka. D) The teleost chromosomes containing many orthologs of the human EGF region were previously shown to be derived from a part of the pre-FSGD teleost protochromosome i , which in turn is derived like Hsa4 from the vertebrate protochromosome C1 .

S5

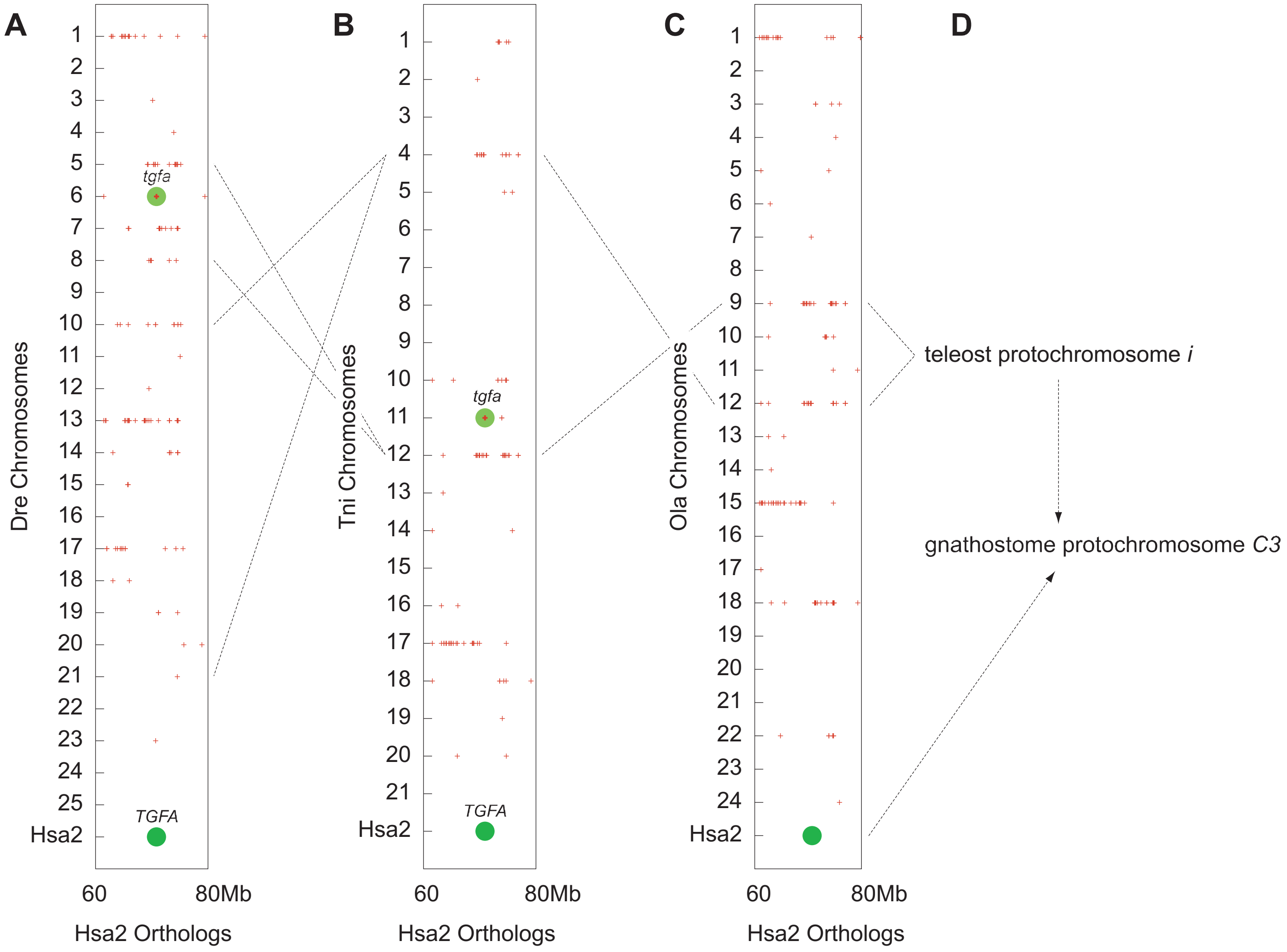

S5) Human TGFA region on Hsa2 in the genomes of A) zebrafish, B) Tetraodon and C) medaka. Several teleost chromosomes, but not the tgfa regions show synteny with the human TGFA region. This may indicate a translocation of the teleost tgfa genes after divergence from tetrapods. D) The teleost chromosomes containing many orthologs of the human TGFA region were previously shown to be derived from a part of the pre-FSGD teleost protochromosome i , which in turn is derived like Hsa2 from the vertebrate protochromosome C3 .

S6

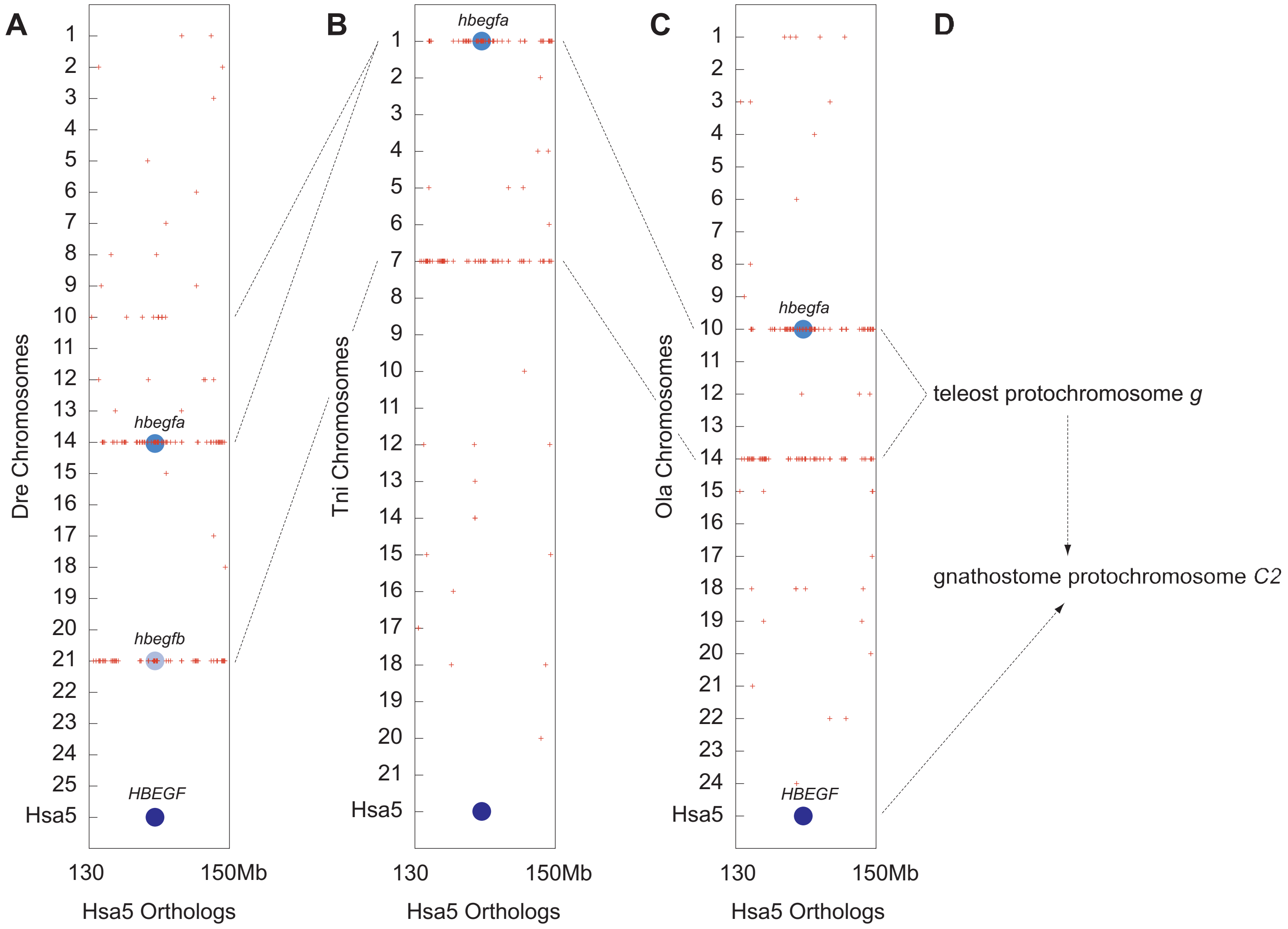

S6) Human HBEGF region on Hsa5 in the genomes of A) zebrafish, B) Tetraodon and C) medaka. D) The teleost chromosomes containing many orthologs of the human HBEGF region, including the two zebrafish hbegf co-orthologs, were previously shown to be derived from the pre-FSGD teleost protochromosome g , which in turn is derived like Hsa5 from the vertebrate protochromosome C2 .

S7

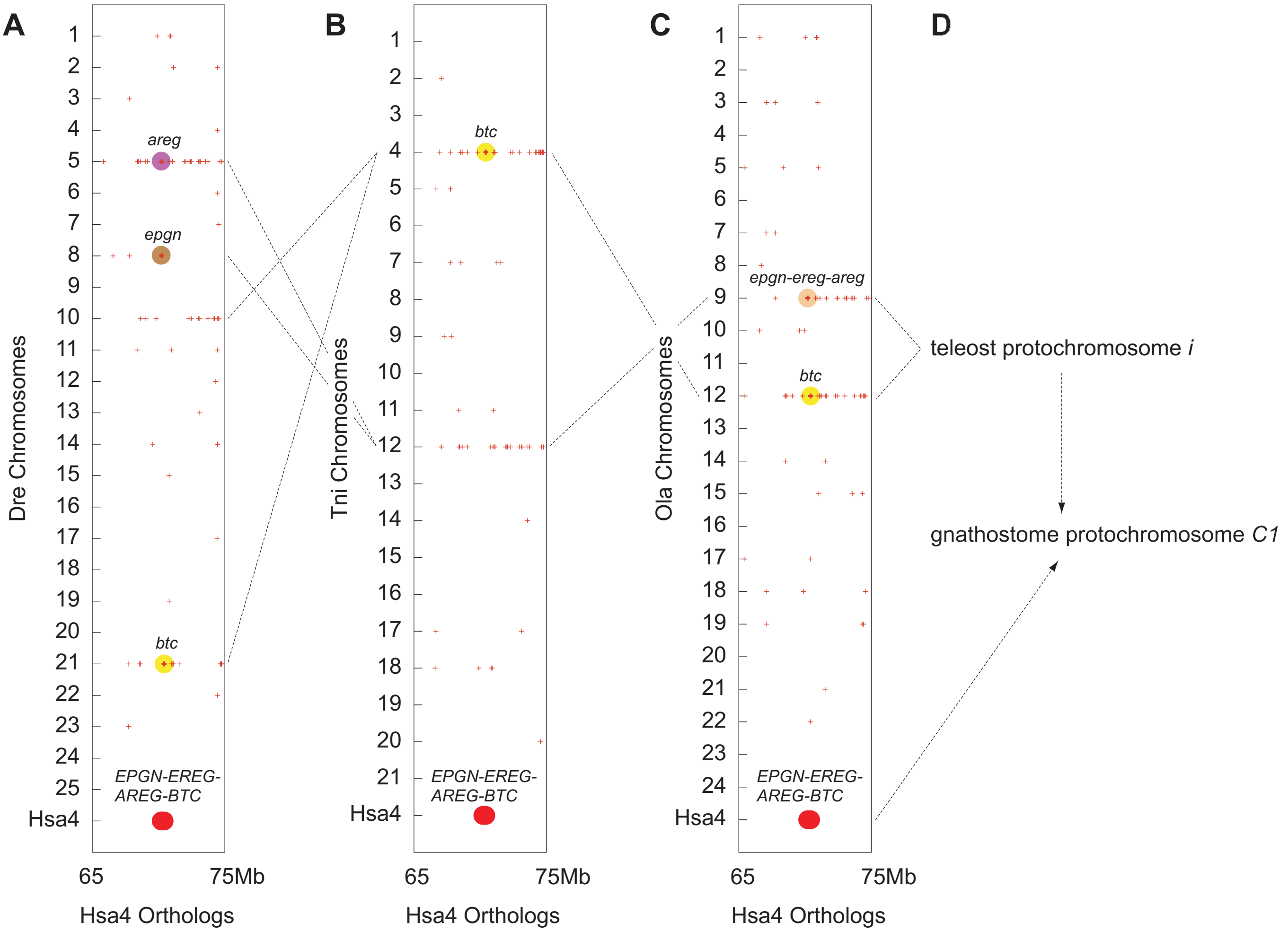

S7) Human ligand cluster region on Hsa4 in the genomes of A) zebrafish, B) Tetraodon and C) medaka. In zebrafish and medaka the btc region and the other ligand gene regions are found on paralogous chromosomes (according to), suggesting cluster breakage by differential genes loss after the FSGD. D) The teleost chromosomes containing many orthologs of the human EGF region were previously shown to be derived from a part of the pre-FSGD teleost protochromosome i , which in turn is derived like Hsa4 from the vertebrate protochromosome C1 .
