# Supplementary material for: Lineage-specific co-evolution of the Egf receptor/ligand signaling system
Source: BMC Evol Biol. 2010 Jan 27;10:27. doi: 10.1186/1471-2148-10-27 (PMC2834686; doi:10.1186/1471-2148-10-27)
Supplement: Additional file 6 — Supplemental table S1. Amino acid similarity between tetrapod and teleost Egf receptor extracellular subdomains. Similarity percentages between human (Hsa), mouse (Mmu), chicken (Gga), medaka (Ola), platyfish (Xma), zebrafish (Dre), green-spotted pufferfish (Tni), fugu (Tru) and three-spined stickleback (Gac) Egfr extracellular subdomains I, II, III and IV. [file 1471-2148-10-27-S6.PDF]

Additional file 6: Supplemental table S1

Amino acid similarity between tetrapod and teleost Egf receptor extracellular subdomains.

Subdomain I	Hsa	Mmu	Gga	Ola	Xma	Dre	Tni	Tru	Gac
Hsa	**100**								
Mmu	85	**100**							
Gga	68	69	**100**						
Ola	54	54	52	**100**					
Xma	53	53	53	79	**100**				
Dre	56	55	55	59	60	**100**			
Tni	47	50	53	70	69	58	**100**		
Tru	47	48	51	74	73	60	85	**100**	
Gac	49	50	49	76	71	60	72	74	**100**

Subdomain II	Hsa	Mmu	Gga	Ola	Xma	Dre	Tni	Tru	Gac
Hsa	**100**								
Mmu	92	**100**							
Gga	82	80	**100**						
Ola	64	64	65	**100**					
Xma	64	65	64	88	**100**				
Dre	67	67	65	75	73	**100**			
Tni	62	63	64	86	87	72	**100**		
Tru	65	65	64	90	89	79	89	**100**	
Gac	64	66	66	89	89	77	86	91	**100**

Subdomain III	Hsa	Mmu	Gga	Ola	Xma	Dre	Tni	Tru	Gac
Hsa	**100**								
Mmu	89	**100**							
Gga	75	73	**100**						
Ola	56	55	58	**100**					
Xma	54	52	57	75	**100**				
Dre	57	54	57	63	60	**100**			
Tni	60	58	58	70	68	61	**100**		
Tru	58	57	61	75	71	63	84	**100**	
Gac	57	55	60	78	69	63	68	63	**100**

Subdomain IV	Hsa	Mmu	Gga	Ola	Xma	Dre	Tni	Tru	Gac
Hsa	**100**								
Mmu	87	**100**							
Gga	64	65	**100**						
Ola	52	51	54	**100**					
Xma	51	47	52	80	**100**				
Dre	51	50	52	65	61	**100**			
Tni	50	50	54	76	72	61	**100**		
Tru	51	51	54	78	74	61	89	**100**	
Gac	50	48	54	76	73	60	76	75	**100**
