# Supplementary material for: Lineage-specific co-evolution of the Egf receptor/ligand signaling system
Source: BMC Evol Biol. 2010 Jan 27;10:27. doi: 10.1186/1471-2148-10-27 (PMC2834686; doi:10.1186/1471-2148-10-27)
Supplement: Additional file 10 — Supplemental tables S2 to S4. S2) List of all species used in this analysis. S3) Accession numbers of all genes used in this analysis. S4) List of primers used for quantitative real-time PCR analyses. [file 1471-2148-10-27-S10.PDF]

Additional file 10: Supplemental table S2

S2) List of all species used in this analysis.

Dre	*D. rerio*	*Danio rerio*	zebrafish
Gac	*G. aculeatus*	*Gasterosteus aculeatus*	three spined stickleback
Gga	*G. gallus*	*Gallus gallus*	chicken
Hsa	*H. sapiens*	*Homo sapiens*	human
Mmu	*M. musculus*	*Mus musculus*	mouse
Ola	*O. latipes*	*Oryzias latipes*	Japanese medaka
Ssa	*S. salar*	*Salmo salar*	Atlantic salmon
Tni	*T. nigroviridis*	*Tetraodon nigroviridis*	pufferfish
Tru	*T. rubripes*	*Takifugu rubripes*	fugu
Xma	*X. maculatus*	*Xiphophorus maculatus*	platyfish

Additional file 10: Supplemental table S3

S3) Accession numbers of all genes used in this analysis.

**Tetrapod *Egfr***	H. sapiens	NM_005228
	M .musculus	NM_207655
	G. gallus	NM_205497
**Teleost *egfra***	*D. rerio*	NM_194424
	G. aculeatus	ENSGACG00000017289
	O. latipes	ENSORLG00000017692
	T. nigroviridis	ENSTNIG00000014840
	T. rubripes	ENSTRUG00000011834
	X.xiphidium	AY230135
**Teleost *egfrb***	*D. rerio*	ENSDARG00000053588
	G. aculeatus	ENSGACG00000018079
	O. latipes	ENSORLG00000003577
	T. nigroviridis	ENSTNIG00000013552
	T. rubripes	ENSTRUG00000017446
	X.xiphidium	X56317
Egf	*H. sapiens*	NM_001963
	M .musculus	NM_010113
	G. gallus	NM_001001292
	D. rerio	NM_205731.1
	G. aculeatus	ENSGACG00000016495
	O. latipes	ENSORLG00000020598
	T. nigroviridis	ENSTNIG00000013026
	T. rubripes	ENSTRUG00000015626
Tgfa	*H. sapiens*	NM_003236
	M .musculus	NM_031199
	G. gallus	NM_001001614
	D. rerio	ENSDARG00000053939
	O. latipes	ENSORLG00000020001
	T. nigroviridis	ENSTNIG00000014720
	T. rubripes	ENSTRUG00000015487
	X.maculatus	GU144294
Areg	*H. sapiens*	NM_001657
	M .musculus	NM_009704
	G. gallus	NM_001031537
	D. rerio	ENSDARG00000031246
	O. latipes	Chromosome 9: 4,580,759-4,584,795
	T. rubripes	scaffold_50: 1,516,226-1,520,278
Btc	H. sapiens	*NM_001729*
	M .musculus	NM_007568
	G. gallus	NM_001004769
	D. rerio	NM_001044764
	O. latipes	ENSORLG00000006542
	T. nigroviridis	ENSTNIG00000007543
	T. rubripes	ENSTRUG00000014500
	X.maculatus	GU144290
Ereg	*H. sapiens*	NM_001044764
	M .musculus	NM_007950
	G. gallus	NM_001001203
	O. latipes	ENSORLG00000001759
	X.maculatus	GU144292
Hb-egf	*H. sapiens*	NM_001945
	M .musculus	NM_010415
	G. gallus	NM_204849
	D. rerio	NM_001100401
	G. aculeatus	ENSGACG00000018355
	O. latipes	ENSORLG00000006983
	T. nigroviridis	ENSTNIG00000008999
	T. rubripes	ENSTRUG00000000850
	X.maculatus	GU144293
Epgn	*H. sapiens*	NM_001013442
	M .musculus	NM_053087
	G. gallus	NM_001012404
	D. rerio	XM_001344355
	O. latipes	ENSORLT00000002186
	X.maculatus	GU144291

Additional file 10: Supplemental table S4

S4) List of primers used for quantitative real-time PCR analyses

	Forward primer	Tm (°C)	Reverse primer	Tm (°C)	Size (bp)
**Ola-*ef1a***	GCCCCTGGACACAGAGACTTCATCA	60,96	AAGGGGGCTCGGTGGAGTCCAT	60,43	**293**
**Ola-*egfra***	ATGGCAGCCCGTTTTCTGAAGTGGATC	61,26	TGATCTCCAGGTTCTCCAGCACCA	59,09	**187**
**Ola-*egfrb***	CATCAGAGGGAGGTCCCTGTTTGTG	60,96	GCCCTTGCTCGAGCACTGCTGG	62,29	**265**
**Ola-*egf***	AAGTTAATCTGGACTGATGTTGGAAGG	56,7	GGGTGTGATTGAAAGCGCTGGCTT	59,09	**170**
**Ola-*tgfa***	TTCGGTCTCACTTTGACGACT	52,4	TAAGAGAGAACACAGCACCATGAAC	56,04	**244**
**Ola-*areg***	GAAGGAGCCGGTGTGTGTAT	53,83	TCCCAAGGTAAGATGCCAAG	51,78	**227**
**Ola-*btc***	CCTGAGGAGCTGACATTTTACTGTG	57,68	TTGGTTCTTCTGTCCTTCTCCTTCT	56,04	**271**
**Ola-*ereg***	GGAGATGGTCCAAGGAAAAATG	52,97	TTAAAGAAGAAGTATGCTGCTCCAG	54,4	**180**
**Ola-*hbegf***	CGATTCTGGAGACTATGAAATGGAC	56,04	CTTGTATTTCTTCAGGCAGGGATT	53,97	**175**
**Ola-*epgn***	GAGTTTGCTTCTCGTCATCACATC	55,67	ACAGAACCCCTCATCCTTACTGTC	57,38	**181**
